# Spatiotemporal gait parameters during turning differ in Parkinson’s disease patients with and without freezing of gait

**DOI:** 10.3389/fnagi.2026.1878271

**Published:** 2026-08-10

**Authors:** Julian Madrid, Baptiste Ulrich-Ischer, Mischa Selig, Brigitte M. Jolles, Julien Favre, David H. Benninger

**Affiliations:** 1 Department of Clinical Neuroscience, Centre Service of Neurology, Lausanne University Hospital and University of Lausanne, Lausanne, Switzerland; 2 Charité–Universitätsmedizin Berlin, Corporate Member of Freie Universität Berlin, Humboldt-Universität zu Berlin, and Berlin Institute of Health, Berlin, Germany; 3 Department of Musculoskeletal Medicine (DAL), Swiss BioMotion Lab, Centre Hospitalier Universitaire Vaudois (CHUV) and University of Lausanne (UNIL), Lausanne, Switzerland; 4 Ecole Polytechnique Fédérale de Lausanne (EPFL), Institute of Microengineering, Lausanne, Switzerland; 5 Department of Orthopedics and Trauma Surgery, G.E.R.N. Research Center for Tissue Replacement, Regeneration & Neogenesis, Faculty of Medicine, Medical Center – University of Freiburg, Freiburg, Germany; 6 The Sense Innovation and Research Center, Lausanne, Switzerland; 7 Department of Neurology, Center for Parkinson and Movement Disorders, Reha Rheinfelden, Rheinfelden, Switzerland; 8 Department of Neurology, University Hospital of Basel, Basel, Switzerland

**Keywords:** freezing of gait, gait, healthy elderly controls, Parkinson’s disease, turning, variability

## Abstract

**Introduction:**

Turning is a complex locomotor task that can accentuate gait impairments and precipitate freezing of gait (FOG) in Parkinson’s disease (PD). However, gait-derived spatiotemporal parameters during inter-episodic turning remain incompletely characterized. We compared turning-related gait patterns among healthy older adults, patients with PD without FOG, and patients with PD and a history of FOG.

**Methods:**

Ten healthy older controls, 10 patients with PD without FOG (PD-P), and 10 patients with PD and FOG (FOG-P) performed 90°, 180°, and 360° turns while undergoing optical motion capture. Spatiotemporal gait parameters were analyzed using Bayesian generalized linear models. Trials containing observable FOG episodes were excluded; consequently, the analyses represent inter-episodic turning rather than freezing episodes themselves.

**Results:**

Compared with controls, PD-P participants demonstrated reduced stride width of the internal leg and increased stride width of the external leg, whereas FOG-P participants demonstrated shorter step and stride lengths. Both PD groups predominantly used step-out or mixed turning strategies and required more time and steps to complete larger-amplitude turns.

**Discussion:**

Gait-derived spatial parameters during turning differed between the PD groups. PD-P participants showed more pronounced mediolateral foot-placement differences, whereas FOG-P participants showed more pronounced reductions in anteroposterior step and stride length. Because the groups differed in overall motor severity and the analyses were intentionally restricted to inter-episodic turning, these findings should be interpreted as descriptive turning gait patterns in patients with a history of FOG rather than as evidence of FOG-specific mechanisms.

## Highlights

- Turning was characterized using gait-derived spatiotemporal parameters across 90°, 180°, and 360° turns.
- Trials with observable FOG episodes were excluded; therefore, findings describe inter-episodic turning in patients with a history of FOG.
- PD patients without FOG showed altered mediolateral foot-placement parameters, particularly stride width.
- PD patients with FOG showed reduced anteroposterior step/stride-length parameters.

## Introduction

Parkinson’s disease (PD) is a progressive neurodegenerative disorder characterized by motor symptoms including bradykinesia, rigidity, tremor, gait impairment, postural instability, and a broad range of non-motor symptoms (Armstrong and Okun, 2020). Although dopaminergic degeneration within the nigrostriatal system is central to PD, gait and balance impairments also reflect dysfunction across distributed cortical, basal ganglia, brainstem, cerebellar, vestibular, and cognitive-attentional networks. Freezing of gait (FOG) is one of the most disabling gait disturbances in PD and is commonly described as brief episodes of absent or markedly reduced forward progression despite the intention to walk. FOG often occurs during gait initiation, turning, passage through narrow spaces, or dual-task walking, and is associated with falls, loss of independence, and reduced quality of life (Gao et al., 2020).

Turning is not simply straight-line gait performed along a curved trajectory. It is a distinct and complex locomotor task requiring anticipatory postural adjustments, dynamic postural control, continuous sensorimotor adaptation, axial rotation, and coordinated reorientation of the head, trunk, pelvis, and feet toward the new direction of travel (Crenna et al., 2007; Mellone et al., 2016). In PD, turning is particularly vulnerable because axial rigidity, impaired automaticity, altered postural control, and increased attentional demands may interfere with the smooth sequencing of body reorientation. Prior studies have shown that PD patients often turn more slowly, take more steps, and use altered turning strategies compared with healthy controls (Stack et al., 2006; Stack and Ashburn, 2008; McNeely and Earhart, 2011; Mellone et al., 2016).

Previous work has also shown that PD patients may use step-out or mixed-turning strategies instead of the crossover strategy more commonly observed in healthy adults (Bhatt et al., 2013). PD patients without FOG have been reported to turn more slowly and with altered step length or step width compared with controls (Crenna et al., 2007; Bhatt et al., 2013), whereas PD patients with FOG often require more time and more steps to turn and may show reduced step length, reduced step width, or increased temporal variability (Spildooren et al., 2010; Bhatt et al., 2013). However, the extent to which gait-derived spatiotemporal parameters during turning differ between PD patients with and without FOG, and whether these differences depend on turning amplitude, remains incompletely understood.

Because turning amplitude influences turning execution and spatiotemporal parameters (Cumming and Klineberg, 1994; Madrid et al., 2023, 2024), a comprehensive description of inter-episodic turning gait in PD and FOG requires studying multiple turning amplitudes. Moreover, variability in spatiotemporal parameters may provide information beyond mean values when characterizing gait alterations in aging and PD (Hausdorff et al., 2001; Galna et al., 2013; Lord et al., 2013).

The objective of this study was therefore to characterize gait-derived spatiotemporal parameters and their variability during 90°, 180°, and 360° turns in healthy elderly controls, PD patients without FOG (PD-P), and PD patients with FOG (FOG-P). By design, trials containing observable FOG episodes were excluded; therefore, the study examines inter-episodic turning gait patterns in patients with a history of FOG rather than the kinematics of FOG episodes themselves. We hypothesized that spatiotemporal parameters during turning would differ between healthy elderly controls, PD-P, and FOG-P, and that these differences would vary across turning amplitudes.

## Materials and methods

### Study participants

The study recruited individuals from three groups: healthy elderly controls, PD patients without freezing of gait (PD-P), and PD patients with freezing of gait (FOG-P), aged between 60 and 85 years. Exclusion criteria were walking impairments unrelated to PD, including musculoskeletal, peripheral nervous, vascular, painful, visual, vestibular, cardiac, respiratory, or metabolic conditions, including obesity (BMI > 30 kg/m^2^), that could independently impair gait. Participants with deep brain stimulation were excluded.

Patients were required to be on stable dopaminergic medication with a total levodopa equivalent daily dose of at least 300 mg/day for at least 1 month. Patients had an OFF-state Hoehn and Yahr stage ranging from 2 to 4 during clinical evaluation. The turning assessments themselves were performed in the ON medication state, defined as testing approximately 45–60 min after the usual dopaminergic medication dose. The medication state was kept uniform across PD-P and FOG-P participants.

FOG-P was defined as a score ≥2 on item 2.13 (“Freezing”) of Part II of the Movement Disorder Society–Unified Parkinson’s Disease Rating Scale (UPDRS) and corroborated by review of clinical files and videotaped trials. Where available, FOG phenotype was classified descriptively from clinical documentation and/or video review as akineto-rigid, trembling, or mixed. These clinical descriptors are reported in Table 1. The study enrolled 30 participants: 10 healthy elderly controls, 10 PD-P, and 10 FOG-P, as shown in Table 1.

The group size of 10 participants per group was determined pragmatically based on the feasibility of recruiting clinically characterized PD-P and FOG-P participants for marker-based motion capture and on prior exploratory studies of spatiotemporal parameters during turning (Bovonsunthonchai et al., 2014; Madrid et al., 2023, 2024). Repeated trials improved within-participant estimation of each participant’s turning pattern, but the number of independent biological observations remained limited to 10 participants per group. Accordingly, the study was designed as an exploratory, descriptive biomechanical study, and between-group results are interpreted cautiously and primarily as descriptive estimates.

**Table 1:** Demographic data.

Demographic data			
Characteristic	Elderly	PD	FOG
Gender (male, female)	4 males	6 males	5 males
Age (years)	72 ± 5	71 ± 7	72 ± 3
Height (m)	1.7	1.7	1.69
BMI (kg/m^2^)	26.4 ± 6.4	23.3 ± 4.2	26.5 ± 2.9
H&Y	NA	2.25 ± 0.26	2.7 **±** 0.48
FOG-Q	NA	2 ± 1	12 ± 4
Clinical Predominant Side (right, left)	NA	6 right	5 right
Subtype akineto-rigid	NA	3	4
Subtype tremor	NA	1	5
Subtype akineto-rigid and tremor	NA	6	1
UPDRS I	NA	12 ± 5	14 ± 9
UPDRS II	NA	11 ± 6	15 ± 9
UPDRS III	NA	21 ± 5	38 ± 13
UPDRS IV	NA	2 ± 2	4 ± 3
UPDRS Total	NA	45 ± 11	71 ± 28

Elderly, healthy elderly controls; PD, Parkinson’s disease patients without freezing of gait; FOG, Parkinson’s disease patients with freezing of gait. BMI, Body mass index; H&Y, Hoehn and Yahr scale; FOG-Q, Freezing of gait questionnaire; UPDRS, unified Parkinson’s disease rating scale.

### Procedure

Participants had reflective markers attached to their feet and pelvis (Ulrich et al., 2019). Foot markers were placed bilaterally on the posterior calcaneus and the second metatarsal heads. Pelvis markers were placed on both anterior superior iliac spines and posterior superior iliac spines.

The pelvis reference system was reconstructed from the four pelvis markers. The origin was defined as the midpoint between the anterior superior iliac spine markers. The mediolateral axis was defined by the line connecting the right and left anterior superior iliac spine markers. The anteroposterior axis was defined perpendicular to the plane formed by the anterior superior iliac spine markers and the midpoint of the posterior superior iliac spine markers. The vertical pelvis axis was obtained as the cross-product of the mediolateral and anteroposterior axes. Pelvis yaw was defined as the rotation of this pelvis reference system around the laboratory vertical axis. Pelvis angular velocity was obtained as the time derivative of the pelvis yaw angle and was processed using the same filtering procedure as marker trajectories.

Participants first performed practice trials and then recorded turning trials barefoot in a laboratory equipped with a 120-Hz optical motion capture system (Vicon, Oxford, UK). Participants were instructed to walk straight for 5 m, execute a 90°, 180°, or 360° turn around a 153-cm vertical rod, and then continue walking straight for at least 3 m toward a predetermined target corresponding to the specific turn (Supplementary Figure S1). Participants were not instructed to use a specific turning strategy, trajectory, or turn sharpness. This was done to allow self-selected turning behavior, but it also means that the turning strategy was not experimentally standardized.

The three turning amplitudes were recorded in randomized order. In healthy elderly controls, turning direction was randomized to the left or right. In PD-P and FOG-P, participants turned toward the clinically more affected side so that the more affected leg became the internal leg during the turn. This choice was made to standardize the clinically relevant side in PD participants, but it introduces an asymmetry between controls and PD groups that is addressed in the limitations. For each turning condition, nine trials were recorded at the participant’s self-selected normal walking speed.

### Data processing

Heel-strike and toe-off events were identified from foot-marker trajectories (Ulrich et al., 2019). Heel-strike detection was based on the method proposed by O’Connor et al. (2007), and toe-off detection was based on the method proposed by Zeni et al. (2008). These approaches were originally developed for straight-line gait and subsequently applied to turning; however, equivalent validation specifically in PD-P and FOG-P during turning has not been established. Therefore, potential gait-event detection errors in patients with shuffling, festination, irregular stepping, or transient motor blocks were considered a methodological limitation. All detected events were visually inspected, and trials with ambiguous heel-strike or toe-off events were excluded from analysis.

Because the computation of spatiotemporal parameters required identifiable heel-strike and toe-off events, trials containing observable FOG episodes were excluded from the analysis. Two independent raters reviewed the videotaped trials and excluded trials in which FOG-P participants manifested FOG episodes. By design, the present analyses characterize inter-episodic turning gait in patients with a history of FOG rather than the spatiotemporal structure of FOG episodes themselves.

The turning period was defined as the portion of the trial during which pelvis angular velocity exceeded 30°/s (Mellone et al., 2016). Extending from this definition, spatiotemporal parameters were analyzed from the last toe-off before the onset of the turning period to the first heel-strike after its end. The following parameters were computed: (1) whole-turn parameters: speed, turning duration, number of steps, and cadence; and (2) internal- and external-leg parameters during each gait cycle: stride length, stride width, step length, gait-cycle duration, stance duration, stance/cycle ratio, and initial double-support duration (Supplementary Figures S1, S2) (Huxham et al., 2006; Perry, 2010).

### Statistical analysis

The reliability of traditional frequentist statistical methods, particularly those involving *p*-values and null hypothesis significance testing (NHST), is currently under scrutiny due to issues with reproducibility (Makowski et al., 2019a; Keysers et al., 2020). The Bayesian approach addresses many of these challenges in a more intuitive manner (Kruschke and Liddell, 2018). A key advantage of the Bayesian method is its ability to distinguish between ‘evidence of absence’ and ‘absence of evidence’ of an effect, a distinction that is not feasible within the frequentist framework (Keysers et al., 2020). We believe this approach is well suited to quantify knowledge and uncertainty about clinical effects regarding the available data. Consequently, we adopted the Bayesian framework for our inferential analysis (Dienes, 2014; Makowski et al., 2019a, 2019b; Keysers et al., 2020). All statistical analyses were conducted using RStudio (version 1.2.1335, RStudio, Inc.).

For every participant involved in the study, data from the nine attempts were pooled for each turning amplitude. Subsequently, for each pooled dataset, the mean and the within-subject standard deviation (SD) were determined to estimate the variability (Hausdorff et al., 2001; Galna et al., 2013). This calculation produced two values for each parameter: one representing the mean and the other the variability, across each specific parameter, turning amplitude, and participant. The standard deviation was preferred to the coefficient of variation (CV) as a measure of variability because the CV can yield misleading results when the values under consideration approach zero (for instance, in the case of stride width).

In order to assess the influence of the disease group (comprising healthy elderly controls, PD-P, and FOG-P) and/or turning amplitude (90°, 180°, 360°) on the spatiotemporal parameters and their variability, statistical models were constructed. These models took into account the disease group as a between-subject factor and the turning amplitude as a within-subject factor, applied separately to the mean and variability values of each parameter. This involved the formulation of a Bayesian generalized linear model incorporating both disease group and turning amplitude as fixed effects, with participant ID serving as a random effect. The disease group and turning amplitude variables were treated as categorical factors in the analysis.

Effect size estimates were determined alongside 95% credible intervals, and standardized effect sizes were calculated as Cohen’s d (Cohen, 1988). Furthermore, we computed the Bayes factor (Dienes, 2014; Makowski et al., 2019a), map-based *p*-values (Mills, 2018; Makowski et al., 2019a), and utilized the Highest Density Interval (HDI) plus Region of Practical Equivalence (ROPE) approach with the standardized effect size. The ROPE was set based on a small effect size (0.2 standard deviations from the data of all parameters) to define the threshold of practical equivalence (Kruschke, 2018). Therefore, this study is not geared to identify very small effect sizes, which we consider to be of limited clinical significance (Cox, 2006).

We utilized residual and q-q plots to verify the independence of data points, absence of unexplained trends in residuals, adherence to linearity and normality, homogeneity of variances, and to identify any outliers. We assessed the convergence of Markov Chain Monte Carlo (MCMC) chains both visually and by applying the Gelman diagnostic. For autocorrelation checks, we relied on visual inspection and ensured that the effective sample size for each parameter was at a minimum of 10,000.

We used weakly informative priors for standardized effect sizes. The primary prior was a normal distribution N(0, 1.6), corresponding to an 80% prior probability that the standardized effect size lies between approximately −2 and 2 (Gronau et al., 2020). This prior was selected to be broad enough for the exploratory aims of the study while avoiding implausibly extreme effects. To assess the influence of prior choice, we performed prior-sensitivity analyses by refitting the main models with a narrower N(0, 0.8) prior and a wider N(0, 2.5) prior. Effects were interpreted as robust only when the direction of the posterior estimate and the HDI-ROPE decision were unchanged across prior specifications.

To gain assurance about the accuracy of our complex model, we represented the actual data mean +/− 95%CI statistics using bootstrapping and the model predictions graphically (Supplementary materials S3–S6).

The significance level was set at a 95% credible Interval on the HDI ROPE of the standardized effect size for significance/equivalence testing.

## Results

**Figure 1:**
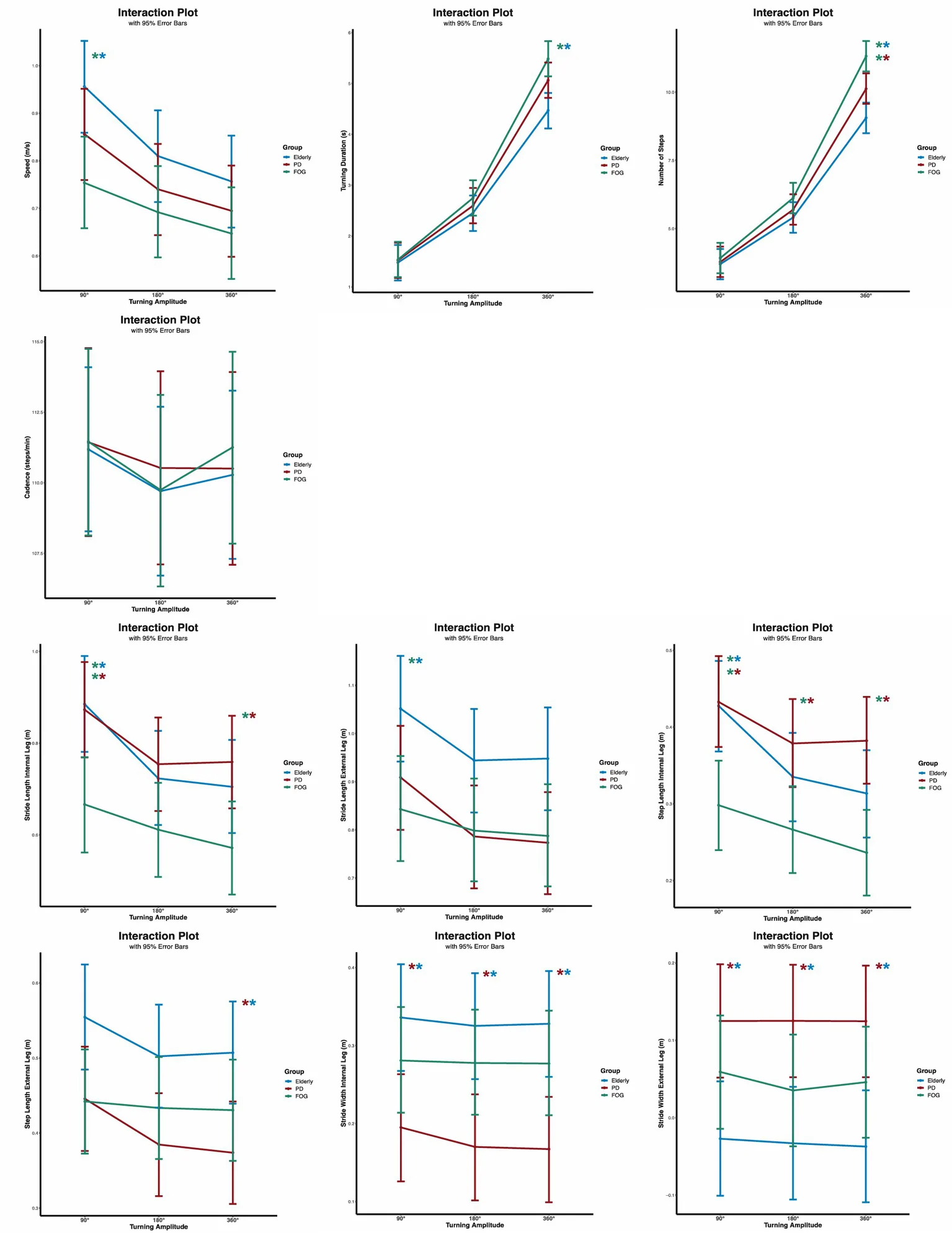
Interaction plots of spatiotemporal and spatial parameters. Interaction plots for model predictions of spatiotemporal and spatial parameters with 95% error bars based on the Bayesian generalized linear model. 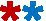 Statistically significant difference between Parkinson’s disease patients without freezing of gait and healthy age matched controls; 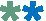 statistically significant difference between Parkinson’s disease patients with freezing of gait and healthy age matched controls; 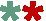 statistically significant difference between Parkinson’s disease patients without freezing of gait and Parkinson’s disease patients with freezing of gait; Elderly, healthy elderly controls age matched individuals; PD, Parkinson’s disease patients without freezing of gait; FOG, Parkinson’s disease patients with freezing of gait.

**Figure 2:**
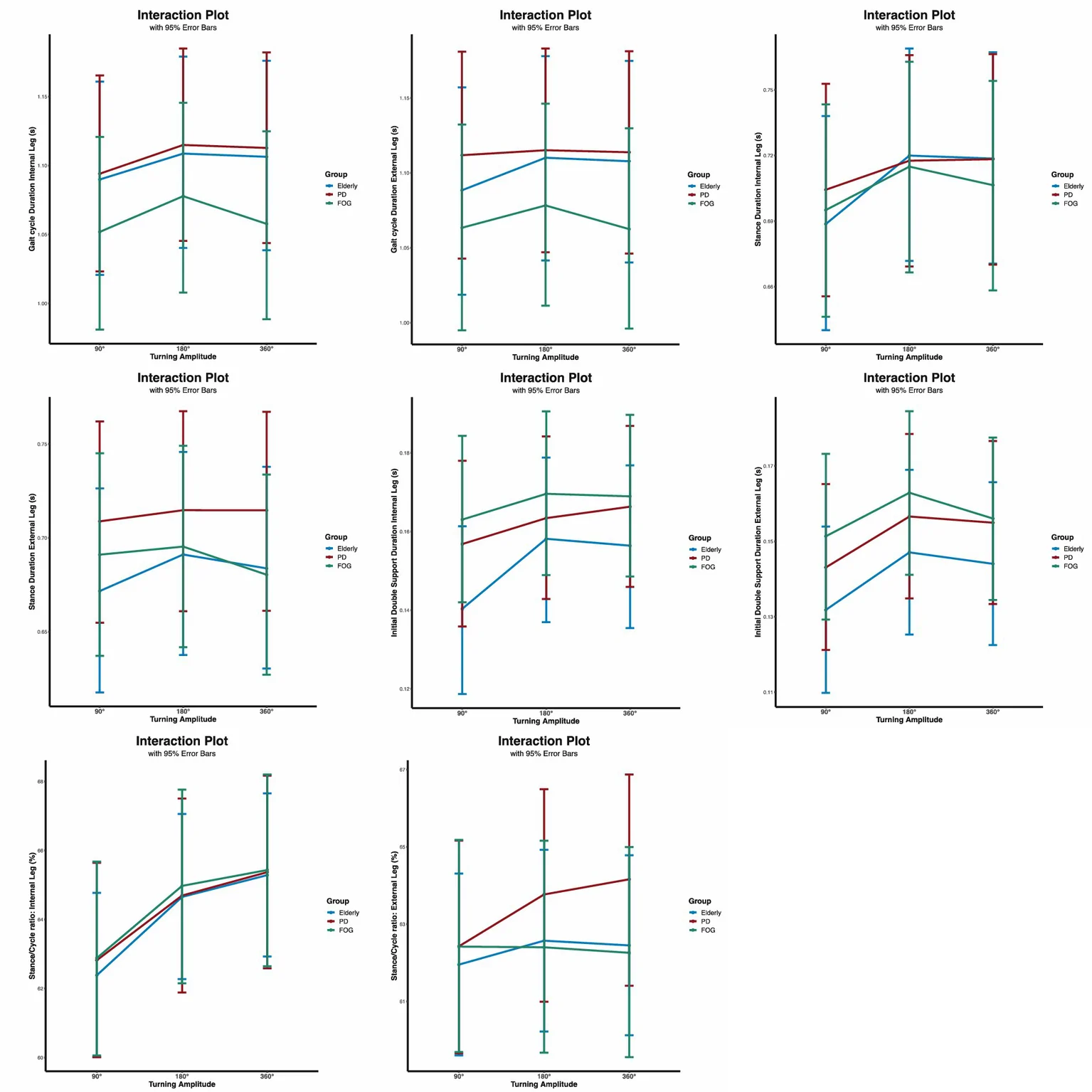
Interaction plots of temporal parameters. Interaction plots for model predictions of temporal parameters with 95% error bars based on the Bayesian generalized linear model. Elderly, healthy elderly age-matched controls; PD, Parkinson’s disease patients without freezing of gait; FOG, Parkinson’s disease patients with freezing of gait.

Demographic and clinical findings are presented in Table 1. The results comparing group effects (healthy elderly controls, PD-P, and FOG-P) and turning-amplitude effects (90°, 180°, and 360°) are summarized in Tables 2, 3 and Figures 1, 2. Two observable FOG episodes (0.25% of all measures), identified at 90° and 180° by two independent raters, were removed before analysis; therefore, all reported results reflect inter-episodic turning. In this section, we present group effects that met the predefined HDI-ROPE criterion. Standardized effect sizes are reported as Cohen’s d with full 95% credible intervals. Exhaustive posterior estimates, Bayes factors, HDI-ROPE decisions, model-predicted means, and full credible intervals are provided in Supplementary materials S3, S5.

**Table 2:** Spatiotemporal parameters.

Parameter	Elderly			PD			FOG			Differences with respect to	
	Mean (95% CI)			Mean (95% CI)			Mean (95% CI)			Group	Turning amplitude
	90°	180°	360°	90°	180°	360°	90°	180°	360°		
Speed (m/s)	0.95	0.808	0.755	0.855	0.74	0.693	0.754	0.692	0.648	90°: E⇔PD⇔FOG | E > FOG	E: 90° > 180° > 360°
	0.925	0.781	0.733	0.82	0.706	0.658	0.718	0.658	0.62	180°: E⇔PD⇔FOG | E⇔FOG	PD: 90° > 180°⇔360° | 90° > 360°
	0.976	0.834	0.778	0.889	0.771	0.726	0.792	0.726	0.676	360°: E⇔PD⇔FOG | E⇔FOG	FOG: 90° > 180°⇔360° | 90° > 360°
Turning Duration (s)	1.488	2.467	4.482	1.52	2.594	5.062	1.546	2.749	5.485	90°: E⇔PD⇔FOG | E⇔FOG	E: 90° < 180° < 360°
	1.419	2.396	4.342	1.453	2.494	4.873	1.479	2.656	5.294	180°: E⇔PD⇔FOG | E⇔FOG	PD: 90° < 180° < 360°
	1.562	2.536	4.632	1.588	2.703	5.25	1.609	2.845	5.725	360°: E⇔PD⇔FOG | E < FOG	FOG: 90° < 180° < 360°
Number of steps	3.756	5.464	9.105	3.761	5.678	10.114	3.92	6.112	11.311	90°: E⇔PD⇔FOG | E⇔FOG	E: 90° < 180° < 360°
	3.622	5.345	8.919	3.636	5.511	9.761	3.782	5.944	10.955	180°: E⇔PD⇔FOG | E⇔FOG	PD: 90° < 180° < 360°
	3.878	5.584	9.302	3.886	5.856	10.421	4.046	6.281	11.689	360°: E⇔PD<FOG | E < FOG	FOG: 90° < 180° < 360°
Cadence (steps/min)	111.512	109.357	109.789	109.531	108.691	108.614	113.532	111.914	113.467	90°: E⇔PD⇔FOG | E⇔FOG	E: 90°⇔180° ≈ 360° | 90° ≈ 360°
	109.214	106.933	107.324	107.044	106.618	106.508	111.925	110.261	112.017	180°: E⇔PD⇔FOG | E⇔FOG	PD: 90° ≈ 180° ≈ 360° | 90° ≈ 360°
	113.85	111.998	112.686	112	110.706	110.67	115.142	113.573	114.996	360°: E⇔PD⇔FOG | E⇔FOG	FOG: 90°⇔180°⇔360° | 90° ≈ 360°
Stride Length	0.877	0.714	0.701	0.852	0.734	0.729	0.654	0.599	0.549	90°: E⇔PD>FOG | E > FOG	E: 90° > 180° ≈ 360° | 90 > 360°
Internal Leg (m)	0.853	0.692	0.686	0.806	0.702	0.707	0.622	0.575	0.535	180°: E⇔PD⇔FOG | E⇔FOG	PD: 90°⇔180° ≈ 360° | 90°⇔360°
	0.902	0.736	0.718	0.893	0.761	0.75	0.687	0.624	0.563	360°: E⇔PD>FOG | E⇔FOG	FOG: 90°⇔180°⇔360° | 90° > 360°
Stride Length	1.041	0.937	0.94	0.912	0.764	0.757	0.858	0.774	0.765	90°: E⇔PD⇔FOG | E > FOG	E: 90° > 180° ≈ 360° | 90° > 360°
External Leg (m)	1.009	0.914	0.927	0.862	0.734	0.736	0.812	0.749	0.746	180°: E⇔PD⇔FOG | E⇔FOG	PD: 90° > 180° ≈ 360° | 90° > 360°
	1.071	0.958	0.954	0.96	0.792	0.78	0.905	0.797	0.783	360°: E⇔PD⇔FOG | E⇔FOG	FOG: 90°⇔180° ≈ 360° | 90°⇔360°
Step Length	0.428	0.332	0.313	0.42	0.369	0.368	0.292	0.262	0.225	90°: E⇔PD>FOG | E > FOG	E: 90° > 180°⇔360° | 90° > 360°
Internal Leg (m)	0.411	0.314	0.303	0.392	0.349	0.354	0.268	0.246	0.217	180°: E⇔PD>FOG | E⇔FOG	PD: 90° > 180° ≈ 360° | 90° > 360°
	0.445	0.35	0.323	0.447	0.391	0.381	0.315	0.276	0.234	360°: E⇔PD>FOG | E⇔FOG	FOG: 90°⇔180°⇔360° | 90° > 360°
Step Length	0.546	0.497	0.502	0.446	0.369	0.364	0.449	0.419	0.418	90°: E⇔PD⇔FOG | E⇔FOG	E: 90° > 180° ≈ 360° | 90° > 360°
External Leg (m)	0.525	0.483	0.493	0.416	0.345	0.349	0.427	0.405	0.409	180°: E⇔PD⇔FOG | E⇔FOG	PD: 90° > 180° ≈ 360° | 90° > 360°
	0.564	0.511	0.51	0.476	0.393	0.381	0.468	0.431	0.428	360°: E > PD⇔FOG | E⇔FOG	FOG: 90° ≈ 180° ≈ 360° | 90° ≈ 360°
Stride Width	0.332	0.322	0.325	0.21	0.177	0.179	0.277	0.272	0.268	90°: E > PD⇔FOG | E⇔FOG	E: 90° ≈ 180° ≈ 360° | 90° ≈ 360°
Internal Leg (m)	0.318	0.315	0.319	0.179	0.155	0.162	0.266	0.264	0.263	180°: E > PD⇔FOG | E⇔FOG	PD: 90°⇔180° ≈ 360° | 90°⇔360°
	0.344	0.329	0.331	0.243	0.199	0.195	0.289	0.28	0.273	360°: E > PD⇔FOG | E⇔FOG	FOG: 90° ≈ 180° ≈ 360° | 90° ≈ 360°
Stride Width	−0.024	−0.031	−0.034	0.132	0.133	0.114	0.057	0.043	0.052	90°: E < PD⇔FOG | E⇔FOG	E: 90° ≈ 180° ≈ 360° | 90° ≈ 360°
External Leg (m)	−0.037	−0.042	−0.041	0.096	0.11	0.097	0.046	0.033	0.045	180°: E < PD⇔FOG | E⇔FOG	PD: 90° ≈ 180° ≈ 360° | 90° ≈ 360°
	−0.009	−0.019	−0.027	0.168	0.158	0.13	0.068	0.053	0.057	360°: E < PD⇔FOG | E⇔FOG	FOG: 90°⇔180° ≈ 360° | 90° ≈ 360°
Gait Cycle Duration	1.087	1.103	1.108	1.082	1.11	1.112	1.057	1.074	1.062	90°: E⇔PD⇔FOG | E⇔FOG	E: 90°⇔180° ≈ 360° | 90°⇔360°
Internal Leg (s)	1.062	1.081	1.093	1.055	1.093	1.099	1.04	1.061	1.054	180°: E⇔PD⇔FOG | E⇔FOG	PD: 90°⇔180° ≈ 360° | 90°⇔360°
	1.111	1.123	1.122	1.11	1.13	1.124	1.073	1.089	1.071	360°: E⇔PD⇔FOG | E⇔FOG	FOG: 90°⇔180°⇔360° | 90° ≈ 360°
Gait Cycle Duration	1.073	1.107	1.106	1.107	1.104	1.108	1.056	1.074	1.064	90°: E⇔PD⇔FOG | E⇔FOG	E: 90°⇔180° ≈ 360° | 90°⇔360°
External Leg (s)	1.049	1.087	1.091	1.081	1.088	1.097	1.039	1.061	1.056	180°: E⇔PD⇔FOG | E⇔FOG	PD: 90° ≈ 180° ≈ 360° | 90° ≈ 360°
	1.098	1.126	1.12	1.135	1.122	1.12	1.072	1.089	1.072	360°: E⇔PD⇔FOG | E⇔FOG	FOG: 90°⇔180°⇔360° | 90° ≈ 360°
Stance Duration	0.687	0.718	0.72	0.702	0.717	0.72	0.698	0.714	0.712	90°: E⇔PD⇔FOG | E⇔FOG	E: 90° < 180° ≈ 360° | 90° < 360°
Internal Leg (s)	0.676	0.706	0.711	0.69	0.707	0.712	0.688	0.706	0.705	180°: E⇔PD⇔FOG | E⇔FOG	PD: 90°⇔180° ≈ 360° | 90°⇔360°
	0.7	0.73	0.729	0.716	0.727	0.727	0.708	0.724	0.719	360°: E⇔PD⇔FOG | E⇔FOG	FOG: 90°⇔180° ≈ 360° | 90°⇔360°
Stance Duration	0.667	0.69	0.684	0.707	0.712	0.714	0.688	0.694	0.684	90°: E⇔PD⇔FOG | E⇔FOG	E: 90°⇔180° ≈ 360° | 90°⇔360°
External Leg (s)	0.655	0.679	0.674	0.692	0.7	0.705	0.678	0.685	0.678	180°: E⇔PD⇔FOG | E⇔FOG	PD: 90° ≈ 180° ≈ 360° | 90° ≈ 360°
	0.68	0.703	0.693	0.724	0.723	0.722	0.698	0.705	0.691	360°: E⇔PD⇔FOG | E⇔FOG	FOG: 90° ≈ 180°⇔360° | 90°⇔360°
Initial double support duration	0.141	0.158	0.158	0.155	0.165	0.169	0.162	0.171	0.173	90°: E⇔PD⇔FOG | E⇔FOG	E: 90° < 180° ≈ 360° | 90° < 360°
Internal Leg (s)	0.136	0.153	0.154	0.149	0.161	0.165	0.155	0.166	0.169	180°: E⇔PD⇔FOG | E⇔FOG	PD: 90°⇔180° ≈ 360° | 90°⇔360°
	0.146	0.163	0.161	0.161	0.17	0.173	0.168	0.176	0.177	360°: E⇔PD⇔FOG | E⇔FOG	FOG: 90°⇔180° ≈ 360° | 90°⇔360°
Initial double support duration	0.132	0.147	0.144	0.142	0.156	0.156	0.152	0.164	0.16	90°: E⇔PD⇔FOG | E⇔FOG	E: 90° < 180° ≈ 360° | 90°⇔360°
External Leg (s)	0.126	0.141	0.14	0.137	0.152	0.153	0.146	0.158	0.157	180°: E⇔PD⇔FOG | E⇔FOG	PD: 90° < 180° ≈ 360° | 90°⇔360°
	0.138	0.152	0.148	0.147	0.162	0.159	0.158	0.17	0.164	360°: E⇔PD⇔FOG | E⇔FOG	FOG: 90°⇔180°⇔360° | 90°⇔360°
Stance/Cycle ratio	62.11	64.525	65.143	62.322	64.055	64.744	64.213	66.205	66.842	90°: E⇔PD⇔FOG | E⇔FOG	E: 90° < 180°⇔360° | 90° < 360°
Internal Leg (%)	61.662	64.117	64.88	61.806	63.638	64.406	63.612	65.839	66.518	180°: E⇔PD⇔FOG | E⇔FOG	PD: 90° < 180°⇔360° | 90° < 360°
	62.531	64.906	65.413	62.873	64.522	65.06	64.808	66.585	67.146	360°: E⇔PD⇔FOG | E⇔FOG	FOG: 90° < 180°⇔360° | 90° < 360°
Stance/Cycle ratio	61.021	61.482	61.428	61.977	63.626	64.066	63.55	64.033	64.045	90°: E⇔PD⇔FOG | E⇔FOG	E: 90°⇔180° ≈ 360° | 90°⇔360°
External Leg (%)	60.592	60.989	61.162	61.218	63.116	63.738	62.933	63.486	63.708	180°: E⇔PD⇔FOG | E⇔FOG	PD: 90° < 180°⇔360° | 90° < 360°
	61.494	61.932	61.699	62.722	64.127	64.413	64.162	64.527	64.366	360°: E⇔PD⇔FOG | E⇔FOG	FOG: 90° ≈ 180° ≈ 360° | 90° ≈ 360°

E, healthy elderly controls; PD, Parkinson’s disease patients without freezing of gait; FOG, Parkinson’s disease patients with freezing of gait. 90°, quarter-turn; 180°, half-turn; 360°, full-turn. >, significantly smaller; <, significantly greater; ≈, equivalent; ⇔, undecided.

**Table 3:** Variability of spatiotemporal parameters.

Parameter SD	Elderly			PD			FOG			Differences with respect to	
	Mean (95% CI)			Mean (95% CI)			Mean (95% CI)			Group	Turning amplitude
	90°	180°	360°	90°	180°	360°	90°	180°	360°		
SD Speed (m/s)	0.0457	0.0381	0.0353	0.056	0.0407	0.033	0.0502	0.0442	0.0332	90°: E⇔PD⇔FOG | E⇔FOG	E: 90°⇔180°⇔360° | 90°⇔360°
	0.0382	0.0329	0.0315	0.0457	0.0331	0.0284	0.0419	0.0363	0.0299	180°: E⇔PD⇔FOG | E⇔FOG	PD: 90° > 180°⇔360° | 90° > 360°
	0.053	0.0442	0.0394	0.0654	0.0479	0.0378	0.0601	0.0524	0.0366	360°: E⇔PD⇔FOG | E⇔FOG	FOG: 90°⇔180°⇔360° | 90° > 360°
SD Turning Duration (s)	0.2706	0.2695	0.3683	0.2938	0.2953	0.3135	0.2926	0.2743	0.3364	90°: E⇔PD⇔FOG | E⇔FOG	E: 90°⇔180°⇔360° | 90°⇔360°
	0.2075	0.2077	0.3094	0.2158	0.2595	0.2845	0.2163	0.2044	0.2531	180°: E⇔PD⇔FOG | E⇔FOG	PD: 90°⇔180°⇔360° | 90°⇔360°
	0.3434	0.3333	0.4537	0.3633	0.3359	0.3463	0.3729	0.3337	0.4307	360°: E⇔PD⇔FOG | E⇔FOG	FOG: 90°⇔180°⇔360°| 90°⇔360°
SD Number of Steps	0.5036	0.4806	0.5901	0.5271	0.5357	0.527	0.5507	0.5092	0.6094	90°: E⇔PD⇔FOG | E⇔FOG	E: 90°⇔180°⇔360° | 90°⇔360°
	0.4138	0.3486	0.5148	0.378	0.499	0.4703	0.3967	0.3617	0.4764	180°: E⇔PD⇔FOG | E⇔FOG	PD: 90°⇔180°⇔360° | 90°⇔360°
	0.6184	0.6046	0.6737	0.6406	0.5784	0.5864	0.6939	0.6173	0.7521	360°: E⇔PD⇔FOG | E⇔FOG	FOG: 90°⇔180°⇔360° | 90°⇔360°
SD Cadence (steps/min)	3.911	3.8526	3.5287	4.2922	3.1299	2.0055	3.9323	2.9576	2.429	90°: E⇔PD⇔FOG | E⇔FOG	E: 90°⇔180°⇔360° | 90°⇔360°
	3.1323	2.5658	2.5757	3.3505	2.2045	1.529	2.7872	2.3646	1.9675	180°: E⇔PD⇔FOG | E⇔FOG	PD: 90°⇔180°⇔360° | 90° > 360°
	4.8711	5.3269	4.5958	5.3431	4.2566	2.6155	4.9995	3.5531	2.9741	360°: E⇔PD⇔FOG | E⇔FOG	FOG: 90°⇔180°⇔360° | 90° > 360°
SD Stride Length	0.0736	0.1097	0.1128	0.0838	0.0858	0.0958	0.0852	0.0864	0.0894	90°: E⇔PD⇔FOG | E⇔FOG	E: 90° < 180°⇔360° | 90° < 360°
Internal Leg (m)	0.0571	0.0962	0.0916	0.0657	0.0662	0.0805	0.0677	0.0754	0.0826	180°: E⇔PD⇔FOG | E⇔FOG	PD: 90°⇔180°⇔360° | 90°⇔360°
	0.0918	0.1252	0.1334	0.108	0.1076	0.1113	0.1019	0.0986	0.0969	360°: E⇔PD⇔FOG | E⇔FOG	FOG: 90°⇔180°⇔360° | 90°⇔360°
SD Stride Length	0.0712	0.0873	0.0701	0.0828	0.0977	0.0813	0.0714	0.0723	0.0731	90°: E⇔PD⇔FOG | E⇔FOG	E: 90°⇔180°⇔360° | 90°⇔360°
External Leg (m)	0.0517	0.0667	0.0611	0.0651	0.0748	0.0677	0.0533	0.0639	0.0616	180°: E⇔PD⇔FOG | E⇔FOG	PD: 90°⇔180°⇔360° | 90°⇔360°
	0.0935	0.1106	0.0791	0.1036	0.1209	0.095	0.0879	0.0805	0.0911	360°: E⇔PD⇔FOG | E⇔FOG	FOG: 90°⇔180°⇔360° | 90°⇔360°
SD Step Length	0.0619	0.0991	0.0828	0.0605	0.0753	0.0629	0.0578	0.0741	0.0635	90°: E⇔PD⇔FOG | E⇔FOG	E: 90° < 180°⇔360° | 90°⇔360°
Internal Leg (m)	0.0496	0.0845	0.0722	0.0465	0.0535	0.0501	0.0429	0.0628	0.0576	180°: E⇔PD⇔FOG | E⇔FOG	PD: 90°⇔180°⇔360° | 90°⇔360°
	0.073	0.1166	0.0951	0.0761	0.0983	0.0775	0.0732	0.0866	0.0695	360°: E⇔PD⇔FOG | E⇔FOG	FOG: 90°⇔180°⇔360° | 90°⇔360°
SD Step Length	0.0475	0.0525	0.0421	0.0522	0.0682	0.0577	0.0405	0.0396	0.0392	90°: E⇔PD⇔FOG | E⇔FOG	E: 90°⇔180°⇔360° | 90°⇔360°
External Leg (m)	0.0349	0.0417	0.0336	0.0406	0.05	0.0476	0.0309	0.0352	0.0328	180°: E⇔PD>FOG | E⇔FOG	PD: 90°⇔180°⇔360° | 90°⇔360°
	0.0595	0.065	0.0515	0.0652	0.0861	0.0683	0.0493	0.0445	0.0469	360°: E⇔PD⇔FOG | E⇔FOG	FOG: 90°⇔180°⇔360° | 90°⇔360°
SD Stride Width	0.0442	0.0377	0.0369	0.0386	0.0375	0.0336	0.0283	0.0249	0.0253	90°: E⇔PD⇔FOG | E⇔FOG	E: 90°⇔180°⇔360° | 90°⇔360°
Internal Leg (m)	0.0376	0.0306	0.0328	0.0276	0.0269	0.0261	0.0216	0.0204	0.0205	180°: E⇔PD⇔FOG | E⇔FOG	PD: 90°⇔180°⇔360° | 90°⇔360°
	0.052	0.0448	0.0407	0.0574	0.053	0.044	0.0371	0.0295	0.0307	360°: E⇔PD⇔FOG | E⇔FOG	FOG: 90°⇔180°⇔360° | 90°⇔360°
SD Stride Width	0.0353	0.0504	0.0449	0.0427	0.0362	0.0368	0.0321	0.0345	0.0399	90°: E⇔PD⇔FOG | E⇔FOG	E: 90° < 180°⇔360° | 90°⇔360°
External Leg (m)	0.0266	0.0406	0.0391	0.0307	0.0283	0.0308	0.0238	0.0282	0.0308	180°: E⇔PD⇔FOG | E⇔FOG	PD: 90°⇔180°⇔360° | 90°⇔360°
	0.0452	0.0612	0.052	0.0579	0.0444	0.0434	0.0403	0.0424	0.0505	360°: E⇔PD⇔FOG | E⇔FOG	FOG: 90°⇔180°⇔360° | 90°⇔360°
SD Gait Cycle Duration	0.0429	0.0678	0.0608	0.0569	0.063	0.0522	0.0425	0.0475	0.0531	90°: E⇔PD⇔FOG | E⇔FOG	E: 90° < 180°⇔360° | 90°⇔360°
Internal Leg (s)	0.0327	0.0521	0.0499	0.0411	0.0458	0.0414	0.0328	0.04	0.0445	180°: E⇔PD⇔FOG | E⇔FOG	PD: 90°⇔180°⇔360° | 90°⇔360°
	0.0529	0.0874	0.071	0.0779	0.08	0.0624	0.0539	0.0557	0.0629	360°: E⇔PD⇔FOG | E⇔FOG	FOG: 90°⇔180°⇔360° | 90°⇔360°
SD Gait Cycle Duration	0.0411	0.0549	0.0522	0.0467	0.0614	0.0488	0.0465	0.0478	0.0441	90°: E⇔PD⇔FOG | E⇔FOG	E: 90°⇔180°⇔360° | 90°⇔360°
External Leg (s)	0.0268	0.0427	0.0409	0.0347	0.0437	0.0371	0.0331	0.0388	0.0347	180°: E⇔PD⇔FOG | E⇔FOG	PD: 90°⇔180°⇔360° | 90°⇔360°
	0.0561	0.0702	0.0632	0.0596	0.0797	0.0611	0.0642	0.0563	0.0559	360°: E⇔PD⇔FOG | E⇔FOG	FOG: 90°⇔180°⇔360° | 90°⇔360°
SD Stance Duration	0.0329	0.0488	0.0466	0.047	0.0497	0.0446	0.0389	0.0394	0.0439	90°: E⇔PD⇔FOG | E⇔FOG	E: 90° < 180°⇔360° | 90° < 360°
Internal Leg (s)	0.0266	0.0388	0.0392	0.0398	0.0397	0.036	0.0322	0.0308	0.036	180°: E⇔PD⇔FOG | E⇔FOG	PD: 90°⇔180°⇔360° | 90°⇔360°
	0.0403	0.0592	0.0543	0.054	0.0583	0.0548	0.0478	0.0467	0.052	360°: E⇔PD⇔FOG | E⇔FOG	FOG: 90°⇔180°⇔360° | 90°⇔360°
SD Stance Duration	0.0319	0.0413	0.041	0.0421	0.0474	0.0412	0.0352	0.0395	0.0361	90°: E⇔PD⇔FOG | E⇔FOG	E: 90° < 180°⇔360° | 90° < 360°
External Leg (s)	0.0251	0.0335	0.033	0.0339	0.036	0.0318	0.0266	0.0323	0.0293	180°: E⇔PD⇔FOG | E⇔FOG	PD: 90°⇔180°⇔360° | 90°⇔360°
	0.039	0.0498	0.0486	0.0516	0.0595	0.0502	0.0438	0.048	0.0433	360°: E⇔PD⇔FOG | E⇔FOG	FOG: 90°⇔180°⇔360° | 90°⇔360°
SD Initial double support duration	0.0149	0.0236	0.0236	0.0195	0.0236	0.0262	0.0181	0.0226	0.0285	90°: E⇔PD⇔FOG | E⇔FOG	E: 90° < 180°⇔360° | 90° < 360°
Internal Leg (s)	0.0118	0.0201	0.0192	0.0141	0.0178	0.0203	0.0144	0.0179	0.023	180°: E⇔PD⇔FOG | E⇔FOG	PD: 90°⇔180°⇔360° | 90°⇔360°
	0.018	0.0271	0.0283	0.0253	0.0302	0.0328	0.0218	0.0285	0.0348	360°: E⇔PD⇔FOG | E⇔FOG	FOG: 90°⇔180°⇔360° | 90° < 360°
SD Initial double support duration	0.0131	0.0191	0.0174	0.0189	0.0239	0.022	0.0172	0.0218	0.0199	90°: E⇔PD⇔FOG | E⇔FOG	E: 90° < 180°⇔360° | 90°⇔360°
External Leg (s)	0.0103	0.0148	0.0149	0.0166	0.0194	0.0178	0.0122	0.0172	0.0161	180°: E⇔PD⇔FOG | E⇔FOG	PD: 90°⇔180°⇔360° | 90°⇔360°
	0.0158	0.0233	0.0202	0.0216	0.0287	0.0261	0.0226	0.0261	0.0239	360°: E⇔PD⇔FOG | E⇔FOG	FOG: 90°⇔180°⇔360° | 90°⇔360°
SD Stance/Cycle ratio	1.4075	1.8984	1.967	1.7844	2.1627	2.084	1.6097	1.8823	2.2082	90°: E⇔PD⇔FOG | E⇔FOG	E: 90°⇔180°⇔360° | 90°⇔360°
Internal Leg (%)	1.1327	1.5961	1.7258	1.3326	1.6527	1.6875	1.0983	1.4805	1.8388	180°: E⇔PD⇔FOG | E⇔FOG	PD: 90°⇔180°⇔360° | 90°⇔360°
	1.7724	2.2312	2.2724	2.2511	2.6814	2.4897	2.1847	2.2492	2.5757	360°: E⇔PD⇔FOG | E⇔FOG	FOG: 90°⇔180°⇔360° | 90° < 360°
SD Stance/Cycle ratio	1.1751	1.8664	1.5563	1.5986	1.7694	1.8713	1.3001	1.7272	1.7026	90°: E⇔PD⇔FOG | E⇔FOG	E: 90° < 180°⇔360° | 90°⇔360°
External Leg (%)	0.9278	1.4775	1.3792	1.169	1.3325	1.5159	0.9041	1.4238	1.4393	180°: E⇔PD⇔FOG | E⇔FOG	PD: 90°⇔180°⇔360° | 90°⇔360°
	1.4201	2.4024	1.7459	2.0818	2.311	2.315	1.7652	2.0383	1.9825	360°: E⇔PD⇔FOG | E⇔FOG	FOG: 90°⇔180°⇔360° | 90°⇔360°

E, healthy elderly controls; PD, Parkinson’s disease patients without freezing of gait; FOG, Parkinson’s disease patients with freezing of gait. 90°, quarter-turn; 180°, half-turn, 360°, full-turn. >, significantly smaller; <, significantly greater; ≈, equivalent; ⇔, undecided.

### Differences between PD patients without FOG (PD-P) and healthy elderly controls

PD-P had a shorter step length of the external leg compared to healthy elderly controls at 360° (Cohen’s d − 2.46 95%CI −4.21, −0.69). PD-P had a decreased stride width of the internal leg compared with healthy elderly controls for all turning amplitudes (Cohen’s d 90°: −4.02 95%CI −6.79, −1.35; Cohen’s d 180°: −4.42 95%CI -7.10, −1.80; Cohen’s d 360°: −4.58 95%CI −7.25, −1.91). PD-P had a significantly increased stride width of the external leg for all turning conditions compared with healthy elderly controls (Cohen’s d 90°: 3.52 95%CI 1.22, 5.80; Cohen’s d 180°: 3.62 95%CI 1.19, 6.00; Cohen’s d 360°: 3.73 95%CI 1.43, 6.03).

We found no significant differences between PD-P and healthy elderly controls in the temporal parameters and the variability of spatiotemporal parameters.

### Differences between FOG patients (FOG-P) and healthy elderly controls

FOG-P walked significantly slower than healthy elderly controls at 90° (Cohen’s d − 3.92 95%CI -6.64, −1.26). At 360° FOG-P needed significantly more time to turn than healthy elderly controls (Cohen’s d 2.18 95%CI 1.34, 3.04). FOG-P needed more steps to turn than healthy elderly controls at 360° (Cohen’s d 2.83 95%CI 2.04, 3.66). FOG-P had a significantly shorter stride length of the internal leg compared with healthy elderly controls at 90° (Cohen’s d − 2.20 95%CI -3.58, −0.85). FOG-P had a shorter stride length of the external leg at 90° compared to healthy elderly controls (Cohen’s d − 2.36 95%CI -4.07, −0.65). FOG-P had a significantly shorter step length of the internal leg compared to healthy elderly controls at 90° (Cohen’s d − 1.65 95%CI -2.66, −0.69).

No significant differences were observed between FOG-P and healthy elderly controls for temporal parameters or for variability of spatiotemporal parameters.

### Differences between FOG patients (FOG-P) and PD patients (PD-P)

FOG-P needed more steps to turn than PD-P at 360° (Cohen’s d 1.73 95%CI 0.96, 2.56). FOG-P had a significantly shorter stride length of the internal leg compared with PD-P at 90° and 360° (Cohen’s d 90°: −2.01 95%CI −3.41, −0.62; Cohen’s d 360°: -1.84 95%CI -−3.19, −0.53). FOG-P had a significantly shorter step length of the internal leg compared to PD-P for all turning amplitudes (Cohen’s d 90°: −1.72 95%CI -−2.69, −0.76; Cohen’s d 180°: −1.43 95%CI −2.37, −0.47; Cohen’s d 360°: −1.85 95%CI −2.81, −0.90).

No significant differences were observed between FOG-P and PD-P for temporal parameters.

FOG-P’s step length of the external leg’s variability was significantly smaller than PD-Ps’ at 180° turning amplitude (Cohen’s d − 1.81 95%CI −3.01, −0.68).

## Discussion

In this study, gait-derived spatiotemporal parameters during inter-episodic turning differed among healthy elderly controls, PD patients without freezing of gait (PD-P), and PD patients with freezing of gait (FOG-P). The main differences were spatial. PD-P showed prominent mediolateral foot-placement alterations, with reduced internal-leg stride width and increased external-leg stride width across turning amplitudes. In contrast, FOG-P showed reduced anteroposterior progression, reflected by shorter step and stride lengths, slower 90° turns, and more time and steps to complete 360° turns. These findings are consistent with previous reports of altered step width in PD and reduced speed, step length, and increased number of steps in FOG-P during turning (Crenna et al., 2007; Spildooren et al., 2010; Bhatt et al., 2013).

Stride-width findings also suggest differences in turning strategy. In healthy elderly controls, negative external-leg stride width was consistent with a crossover strategy. In contrast, positive external-leg stride width in PD-P and FOG-P was consistent with step-out or mixed turning strategies, as previously described in PD (Bhatt et al., 2013; Conradsson et al., 2018). None of the groups clearly increased stride width with turning amplitude, suggesting that larger turns were accommodated mainly through additional steps, longer turning duration, or altered foot placement rather than by systematically widening the base of support.

The distinction between mediolateral and anteroposterior parameters may be clinically relevant. PD-P differed most consistently in stride-width parameters, whereas FOG-P differed most consistently in step- and stride-length parameters, particularly for the internal, more affected leg. Operationally, stride width reflects mediolateral foot-placement geometry, whereas step and stride length reflect forward progression during the turn. However, these variables should not be interpreted as isolated mechanisms. They may reflect interacting effects of bradykinesia, axial rigidity, impaired postural control, attentional demand, cautious gait, disease severity, and self-selected turning strategy. Because the FOG-P group had higher UPDRS III scores than the PD-P group, differences between PD-P and FOG-P cannot be attributed uniquely to FOG.

Turning amplitude influenced the observed group differences. Several step- and stride-length differences were most evident during 90° turns, whereas stride-width differences were present across turning amplitudes. This suggests that smaller, everyday turns may be particularly informative for detecting anteroposterior progression deficits, whereas mediolateral foot-placement alterations may be less dependent on turn amplitude. This finding may be useful for future protocol design, especially because 90° turns are frequent in daily life, but it requires confirmation in larger cohorts.

Only one group-related variability difference was observed: external-leg step-length variability was lower in FOG-P than in PD-P during 180° turns. This isolated finding should be interpreted cautiously. Contrary to straight-line gait studies in which increased variability has been associated with disease progression, fall risk, or FOG (Hausdorff et al., 2001, 2003; Mbourou et al., 2003), reduced variability during turning could reflect limited adaptive capacity, a cautious strategy, or task-specific constraints. The present data do not allow these alternatives to be distinguished.

Overall, these findings support the view that turning has a spatiotemporal signature that differs from straight-line walking. Straight-line gait studies in PD commonly report changes in speed, cadence, stride length, and double-support duration, whereas studies in FOG often emphasize reduced step length and increased stride-length variability (Hausdorff et al., 2003; Nanhoe-Mahabier et al., 2011; Zanardi et al., 2021). In the present turning task, the most consistent abnormalities were spatial, particularly mediolateral stride-width changes in PD-P and anteroposterior step- and stride-length reductions in FOG-P. Consistent with the study scope, these results describe inter-episodic turning patterns rather than the biomechanics of freezing itself.

From a neurophysiological perspective, turning requires locomotor automaticity, axial coordination, postural control, and attentional resources. Reduced step and stride length in FOG-P may reflect impaired amplitude scaling or cautious progression, whereas altered stride width in PD-P may reflect changes in mediolateral foot-placement strategy. These interpretations remain hypotheses because neural activity, axial coordination, anticipatory postural adjustments, and center-of-mass dynamics were not directly measured. Nevertheless, the present results may help generate rehabilitation hypotheses, including whether training focused on step amplitude, turning strategy, and anteroposterior progression can improve turning in PD patients with FOG, potentially in combination with neuromodulatory approaches such as non-invasive brain stimulation (Madrid and Benninger, 2021; Madrid et al., 2026).

### Limitations

Several limitations should be considered. First, this study quantified gait-derived spatiotemporal parameters during turning, not comprehensive turning kinematics or motor-control mechanisms. Head–trunk–pelvis coordination, axial rigidity, center-of-mass control, anticipatory postural adjustments, and dynamic stability were not directly assessed. Second, gait events were detected using kinematic methods originally developed for straight-line gait and subsequently applied to turning. Although all events were visually inspected and ambiguous trials were excluded, these methods have not been specifically validated for PD-P and FOG-P during turning. Shuffling, festination, irregular stepping, asymmetry, and transient motor blocks may therefore have influenced event detection and downstream parameter estimates.

Third, the sample size was limited to 10 participants per group. Repeated trials improved within-participant estimates, but they did not increase the number of independent biological observations. The Bayesian framework provided structured uncertainty estimation, but it cannot compensate for the limited sample size. These findings should therefore be considered exploratory.

Fourth, group comparisons may be confounded by clinical and methodological differences. The FOG-P group had higher UPDRS III scores than the PD-P group; turning strategy, trajectory, and turn sharpness were self-selected; and controls turned in randomized directions whereas PD-P and FOG-P turned toward the clinically more affected side. Spatial parameters were analyzed in absolute units rather than normalized to body height or leg length. Height was recorded and was similar across groups (Table 1), but leg length was not directly measured as a standardized anthropometric variable, and marker-derived distances from dynamic trials were not considered equivalent to standardized leg-length measurements. We retained absolute spatial parameters to remain consistent with the original processing pipeline and prior turning studies using the same parameter definitions. Nevertheless, because body-height or leg-length normalization was not performed, residual anthropometric confounding cannot be excluded, particularly for step length, stride length, and stride width. Spatial group differences should therefore be interpreted as absolute gait-derived differences rather than body-size-normalized effects.

Finally, the levodopa responsiveness of turning parameters and FOG, FOG phenotype, and cognitive performance were not systematically assessed. Data-reduction methods such as principal component analysis were also not applied because of the small sample size. Future studies should include larger cohorts, anthropometric normalization or normalized sensitivity analyses, cognitive testing, medication-state comparisons, FOG-phenotype characterization, formal turning-strategy analysis, and additional biomechanical measures.

## Conclusion

In summary, gait-derived spatiotemporal parameters during turning differed between healthy elderly controls, PD-P, and FOG-P. PD-P showed mainly mediolateral foot-placement alterations, whereas FOG-P showed mainly reduced anteroposterior step and stride length. By design, these findings describe inter-episodic turning patterns in patients with a history of FOG rather than the biomechanics of freezing itself. Interpretation is limited by the small sample size, greater motor severity in FOG-P, self-selected turning strategies, and the absence of anthropometric normalization and cognitive characterization. Larger studies combining spatiotemporal parameters with axial kinematics, dynamic stability measures, medication-state assessment, and FOG-phenotype characterization are needed to clarify turning-specific impairments in PD and FOG.

## Data Availability

The raw data supporting the conclusions of this article will be made available by the authors, without undue reservation.

## Supplementary material

SUPPLEMENTARY FIGURE S1 Experimental turning setup. Illustration of the experimental procedure, with turns of 90° **(a)**, 180° **(b)** and 360° **(c)**. The colors indicate internal versus external foot placement. The numbers indicate the order of the foot placements and start at turning initiation. Adapted from Madrid et al. (2023) with permission.

SUPPLEMENTARY FIGURE S2 Definition of spatiotemporal parameters. **(a)** Spatial gait parameters: stride length of internal leg (StrLI); stride length of external leg (StrLE); step length of internal leg (SteLI); step length of external leg (SteLE); stride width of internal leg (SWI); stride width of external leg (SWE). **(b)** Temporal gait parameters: internal heel-strike event (IHS); external toe-off event (ETO); external heel-strike event (EHS); internal toe-off event (ITO); gait cycle duration of the internal leg (GCI); gait cycle duration of the external leg (GCE); stance duration of the internal leg (SDI); stance duration of the external leg (SDE); initial double-support duration of the internal leg (IDbI); initial double-support duration of the external leg (IDbE). Adapted from Madrid et al. (2023) with permission.

SUPPLEMENTARY MATERIAL S3 Spatiotemporal parameters. Exhaustive results for spatiotemporal parameters.

SUPPLEMENTARY MATERIAL S4 Spatiotemporal parameters. Figure 1: Parallel coordinates Plot of spatiotemporal parameters; Figure 2: Interaction Plot for the actual data and bootstrapped uncertainty estimates; Figure 3: Interaction plot for model predictions.

SUPPLEMENTARY MATERIAL S5 Spatiotemporal parameters’ variability. Exhaustive results for spatiotemporal parameters’ variability.

SUPPLEMENTARY MATERIAL S6 Spatiotemporal parameters’ variability. Figure 1: Parallel coordinates Plot of spatiotemporal parameters’ variability; Figure 2: Interaction Plot for the actual data and bootstrapped uncertainty estimates; Figure 3 Interaction plot for model predictions.
