# Supplementary material for: Spatiotemporal gait parameters during turning differ in Parkinson’s disease patients with and without freezing of gait
Source: Front Aging Neurosci. 2026 Aug 10;18:1878271. doi: 10.3389/fnagi.2026.1878271 (PMC13500623; doi:10.3389/fnagi.2026.1878271)
Supplement: Supplementary file 1 [file Data_Sheet_1.pdf]

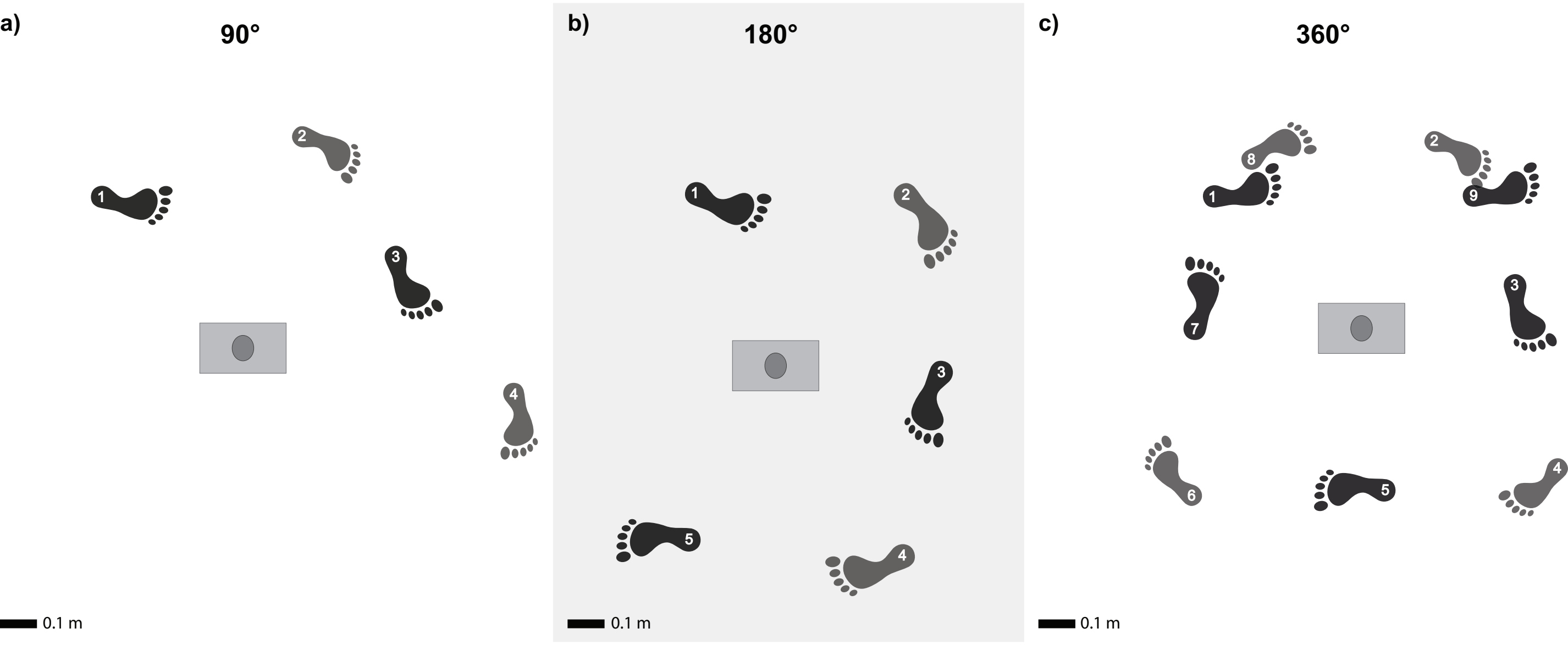
a)
90°
1
2
3
4
0.1 m
b)
180°
1
2
3
4
5
0.1 m
c)
360°
1
2
3
4
5
6
7
8
9
0.1 m
