# Supplementary material for: Spatiotemporal gait parameters during turning differ in Parkinson’s disease patients with and without freezing of gait
Source: Front Aging Neurosci. 2026 Aug 10;18:1878271. doi: 10.3389/fnagi.2026.1878271 (PMC13500623; doi:10.3389/fnagi.2026.1878271)
Supplement: Supplementary file 2 [file Data_Sheet_2.pdf]

a)

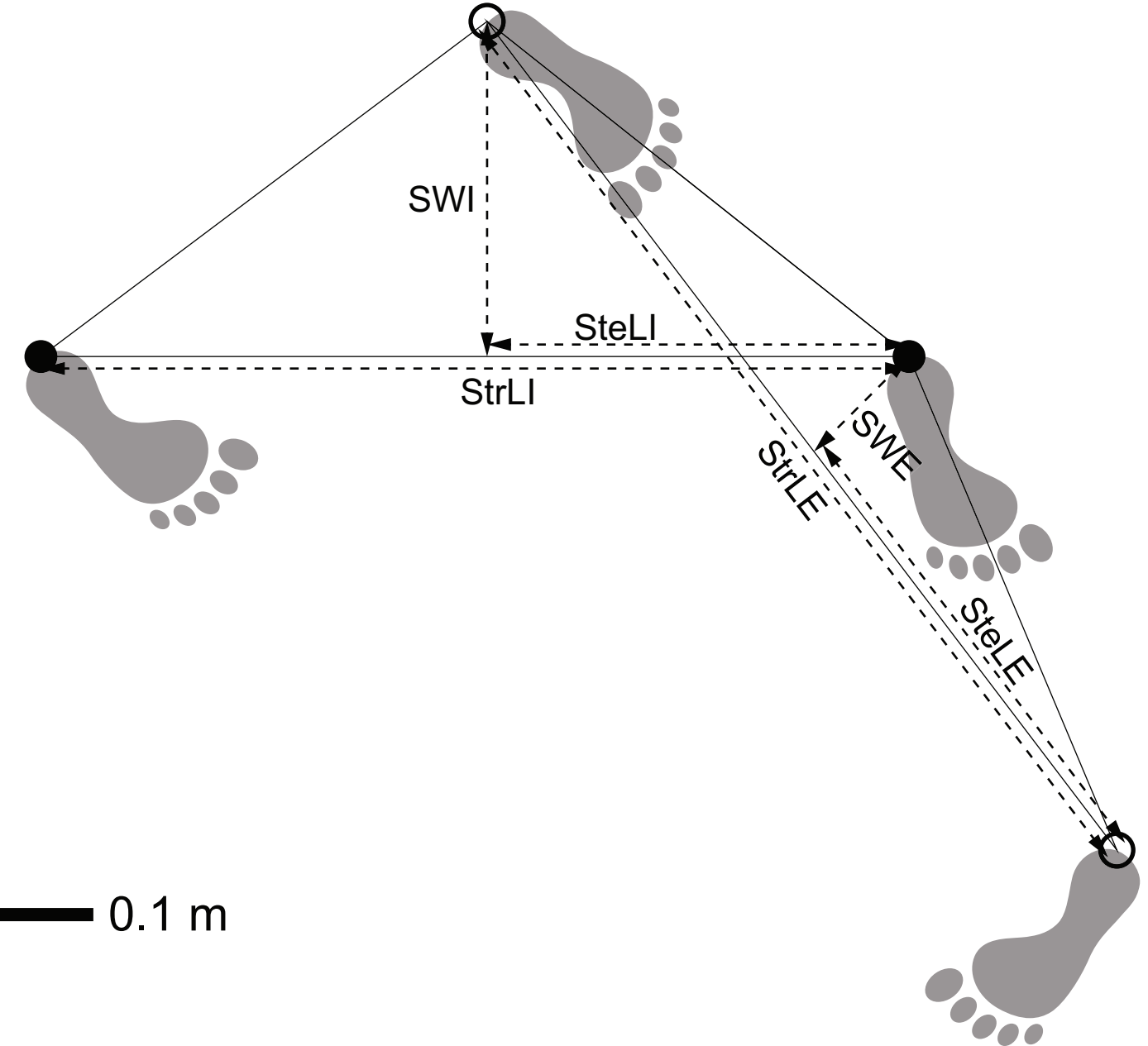
SWI
SteLI
StrLI
SWE
StrLE
SteLE
0.1 m

b)

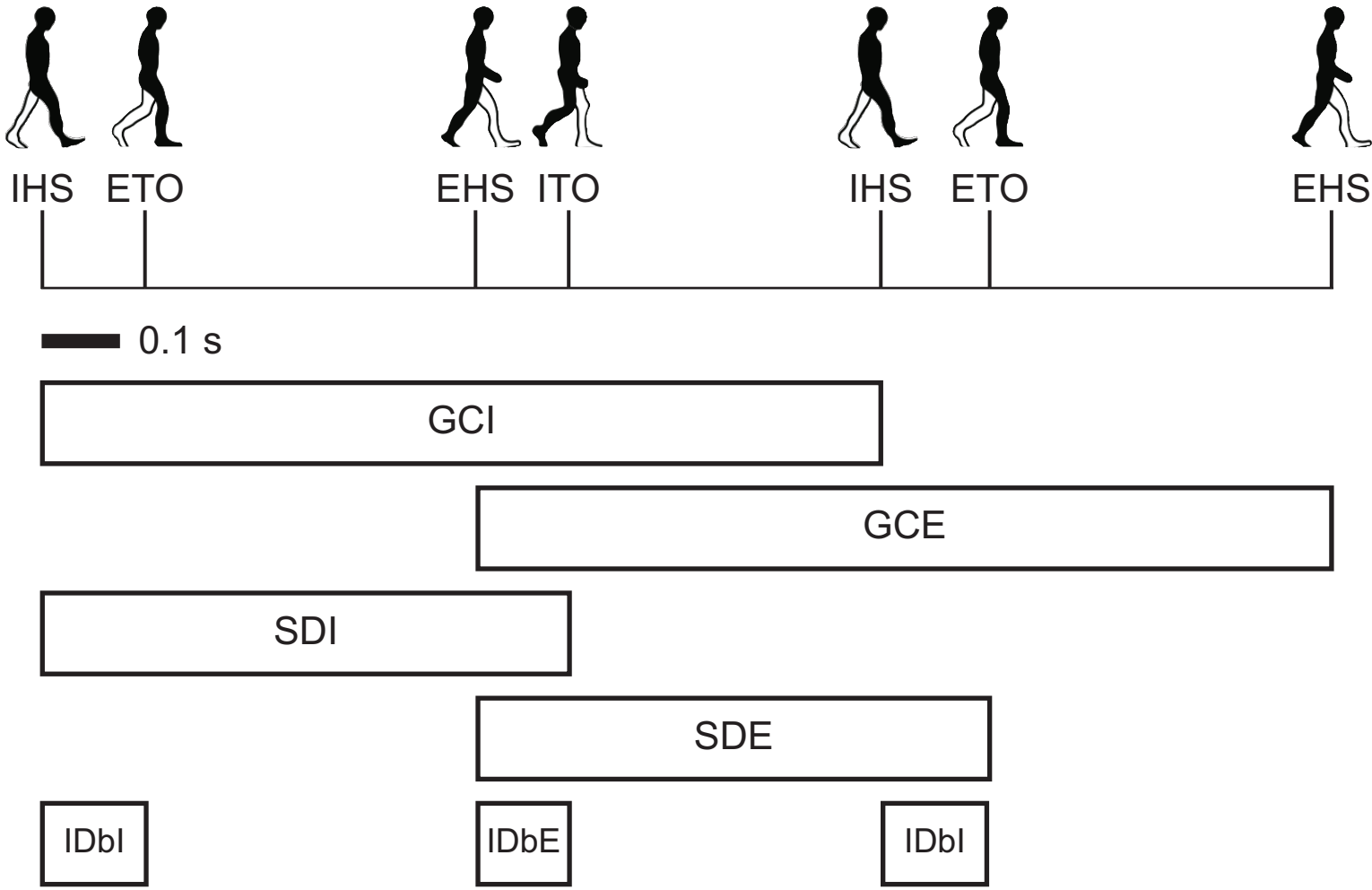
IHS
ETO
EHS
ITO
IHS
ETO
EHS
0.1 s
GCI
GCE
SDI
SDE
IDbI
IDbE
IDbI
