# Supplementary material for: Spatiotemporal gait parameters during turning differ in Parkinson’s disease patients with and without freezing of gait
Source: Front Aging Neurosci. 2026 Aug 10;18:1878271. doi: 10.3389/fnagi.2026.1878271 (PMC13500623; doi:10.3389/fnagi.2026.1878271)

Figure 1

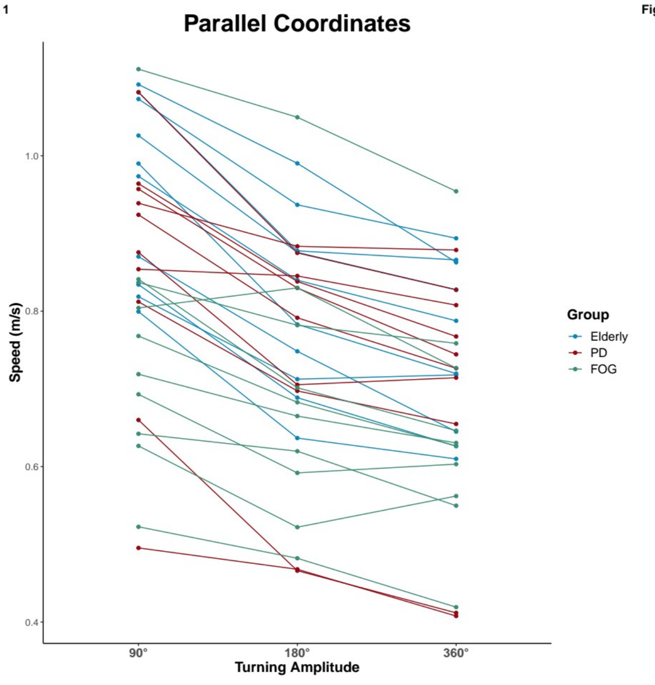


Figure 2

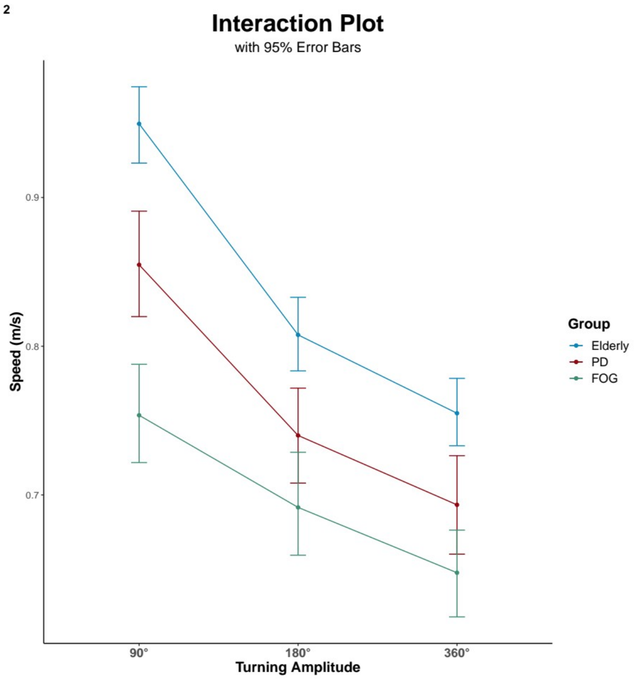


Figure 3

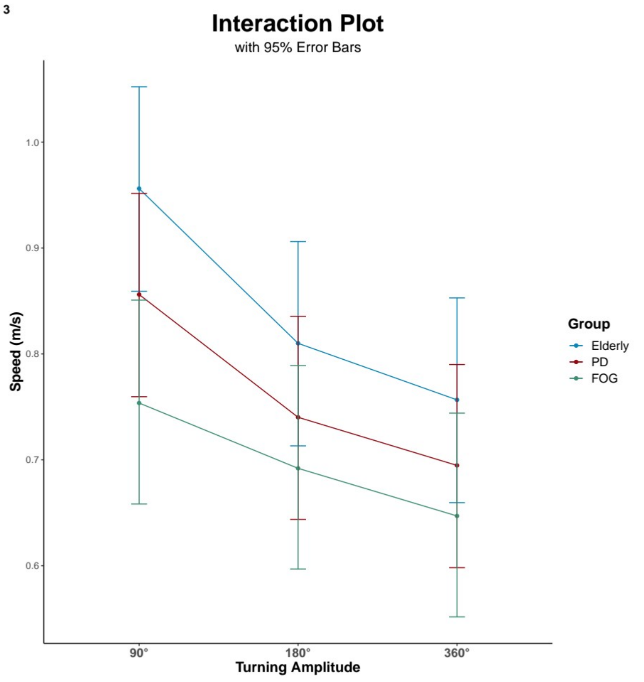

Figure 1

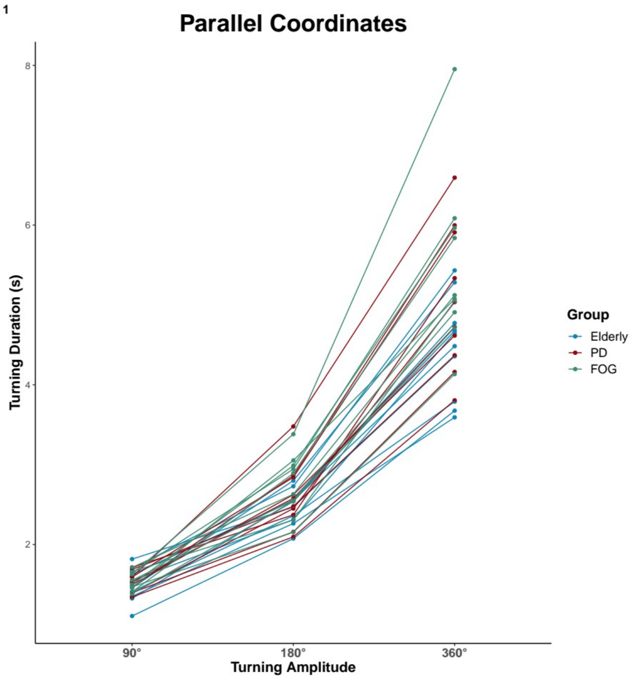


Figure 2

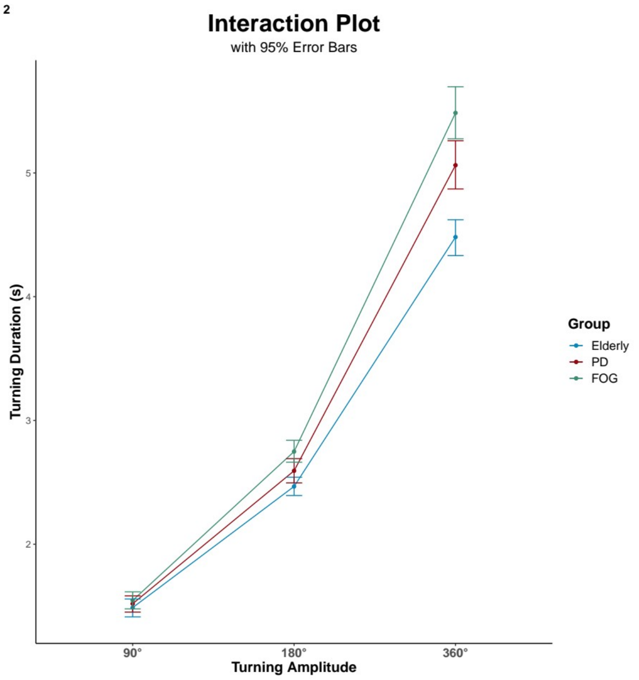


Figure 3

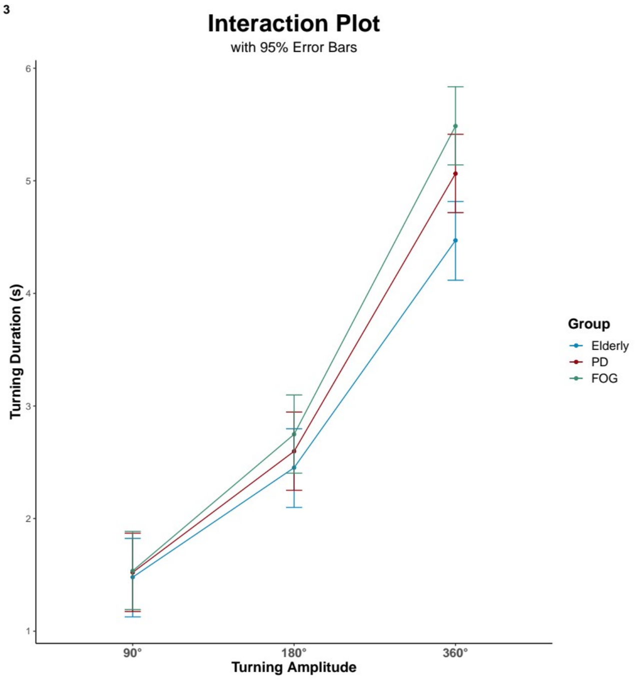

Figure 1

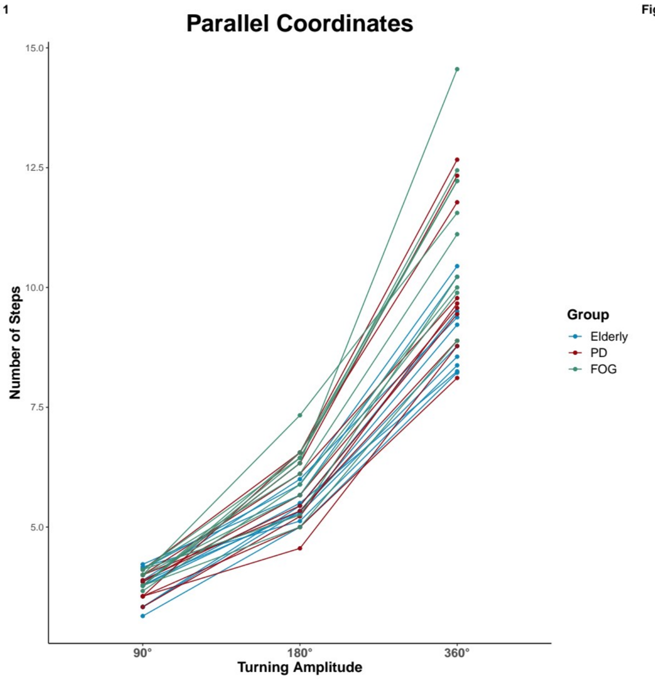


Figure 2

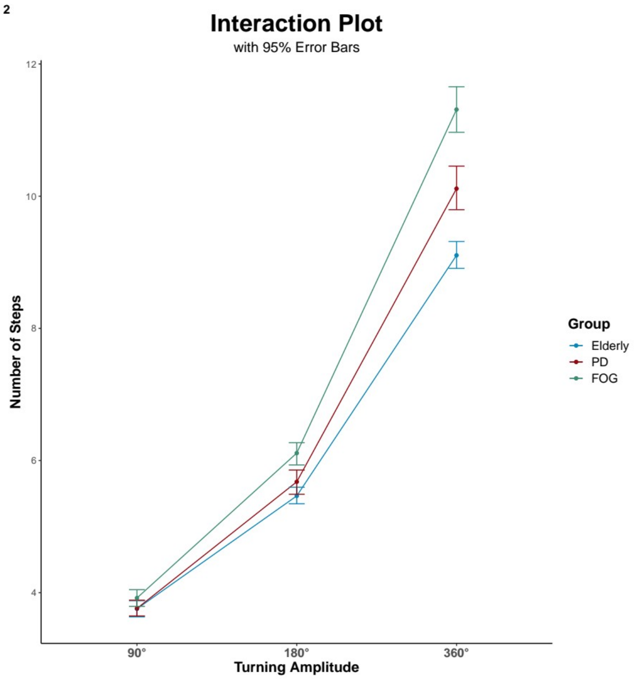


Figure 3

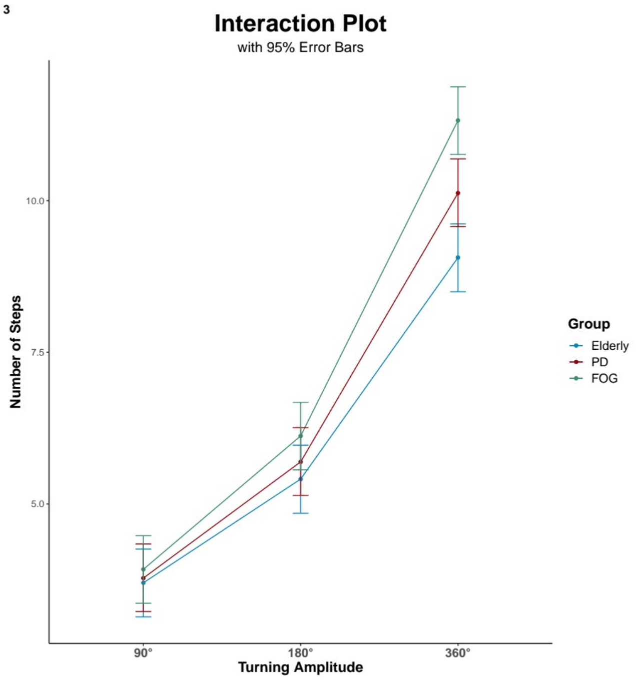

**Figure 1**

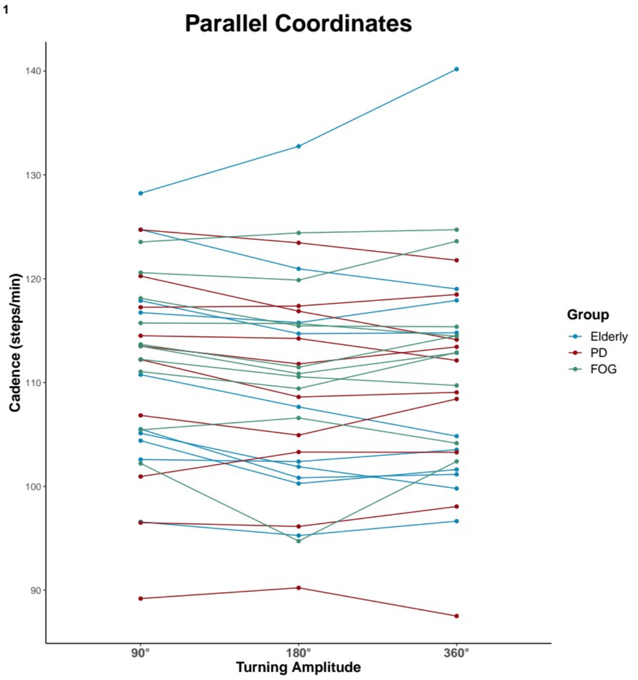


**Figure 2**

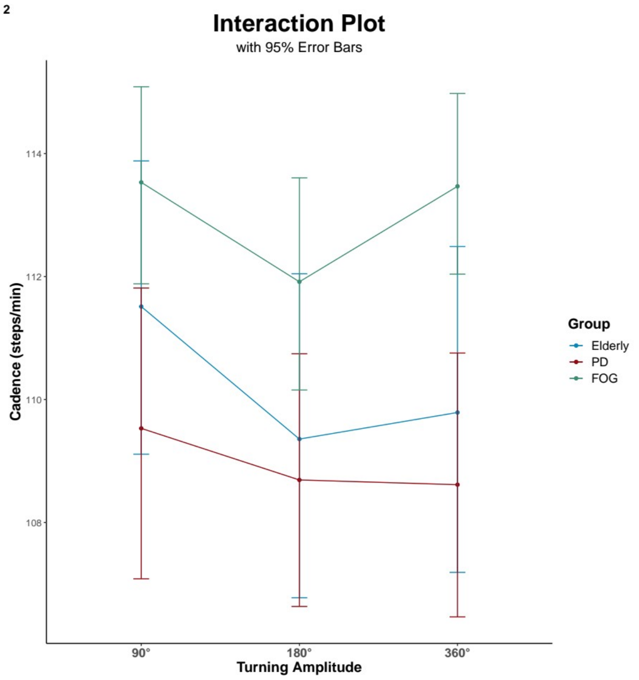


**Figure 3**

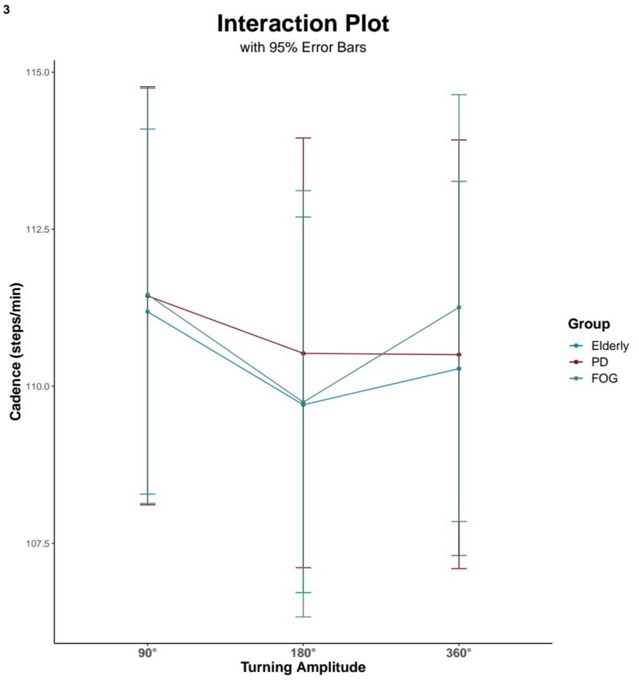

Figure 1

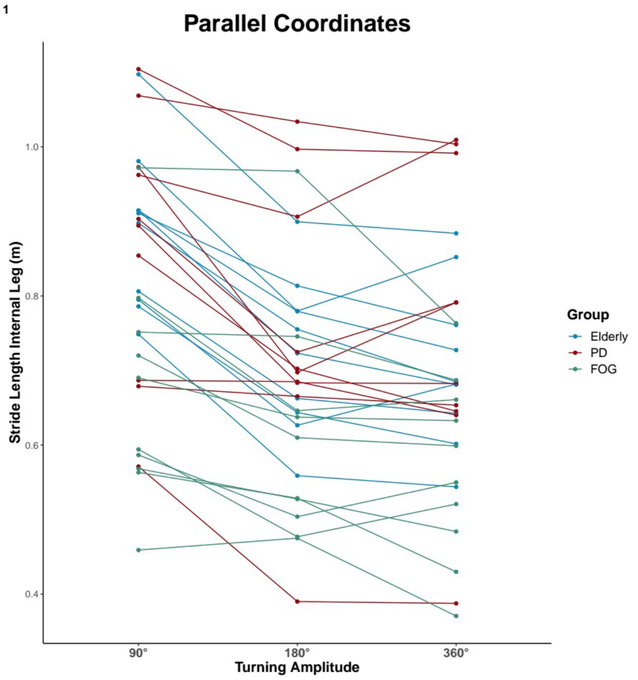


Figure 2

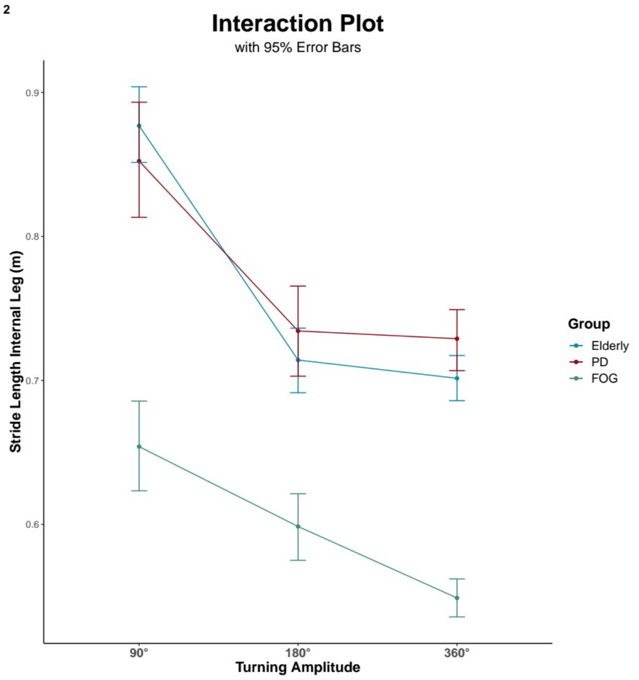


Figure 3

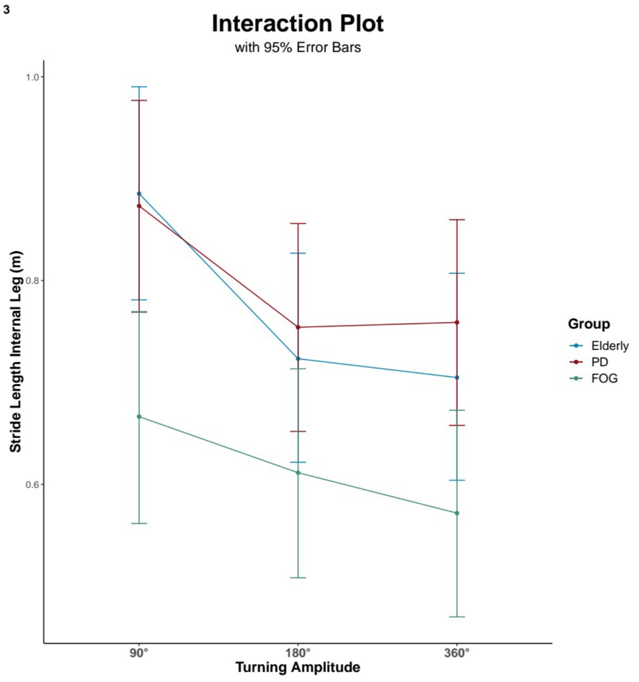

Figure 1

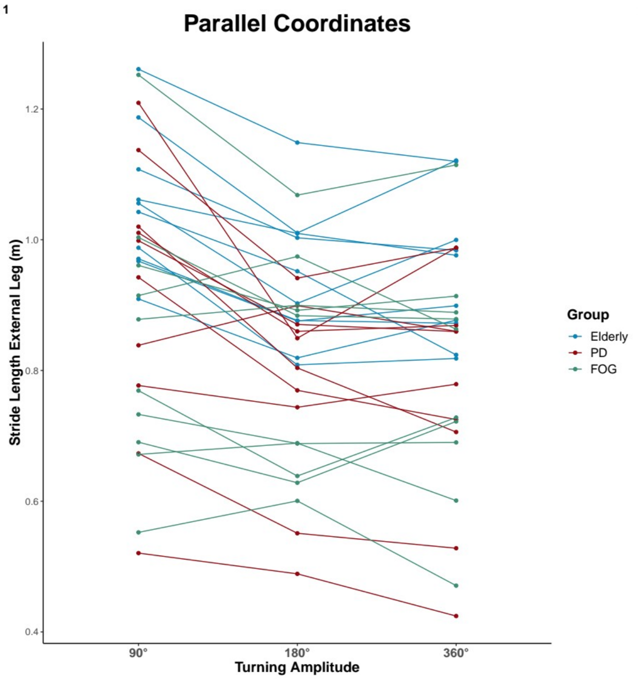


Figure 2

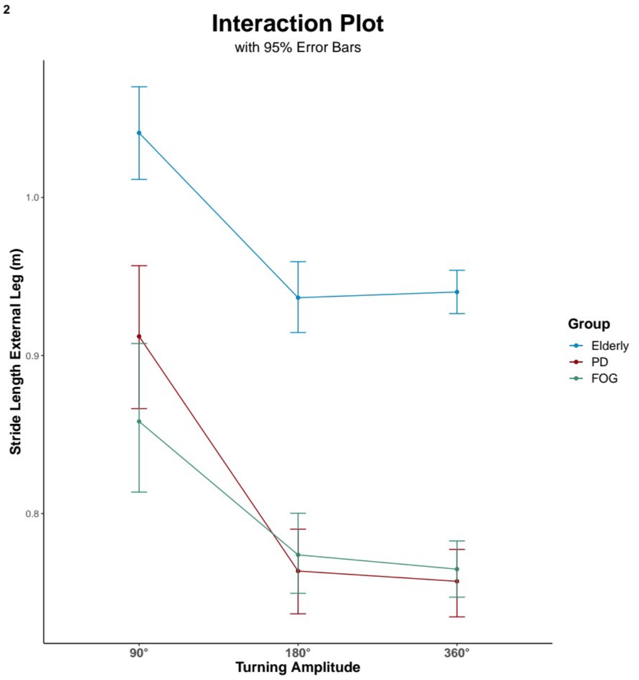


Figure 3

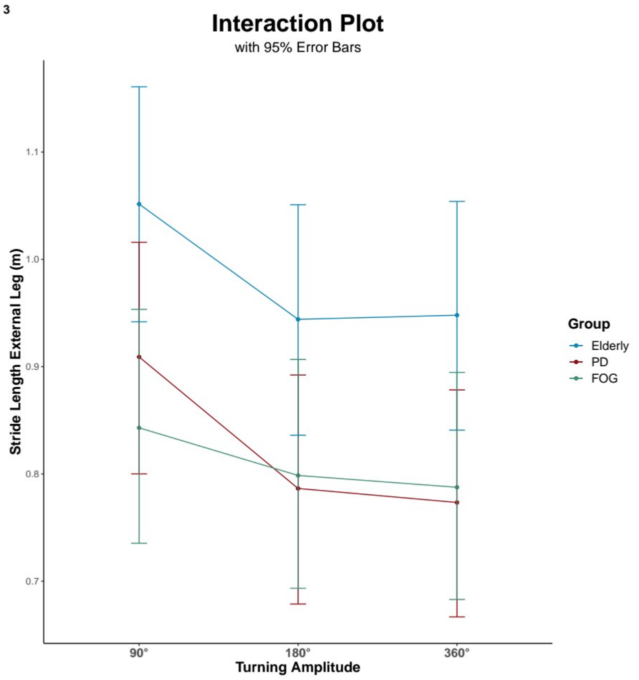

Figure 1

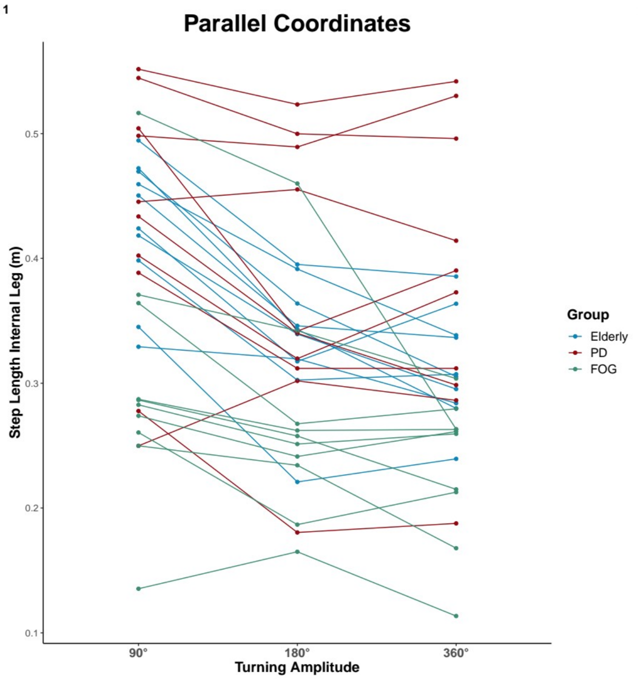


Figure 2

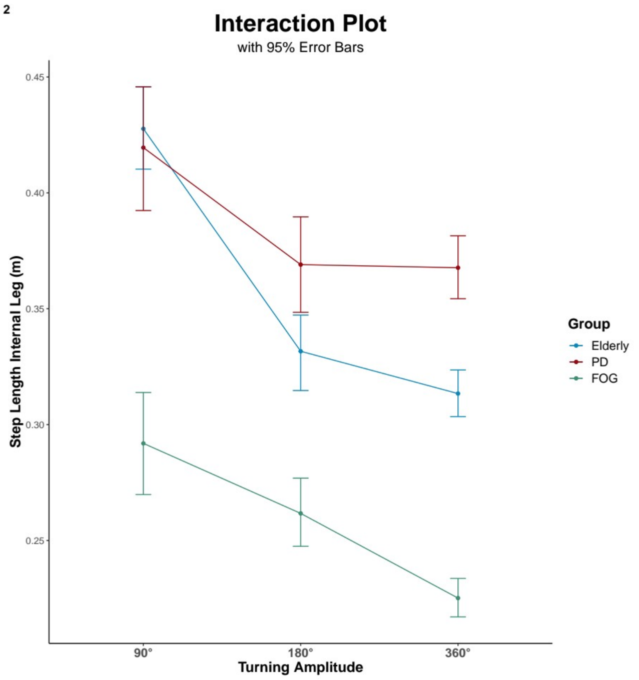


Figure 3

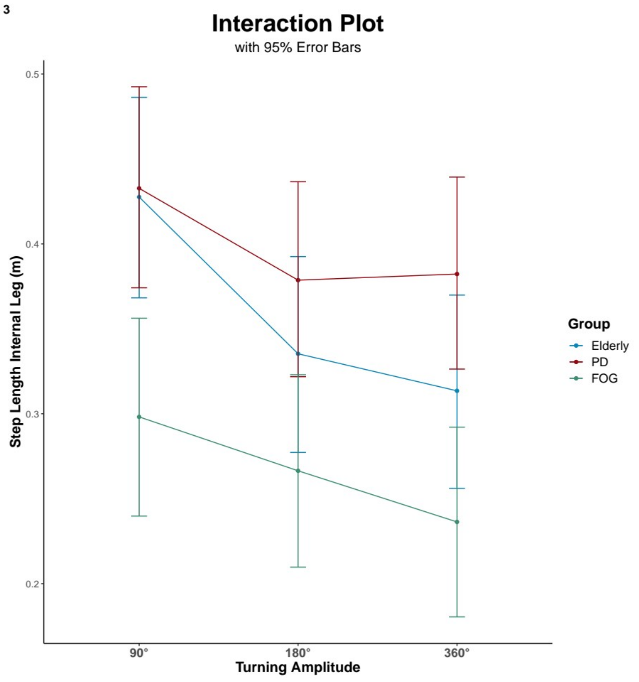

Figure 1

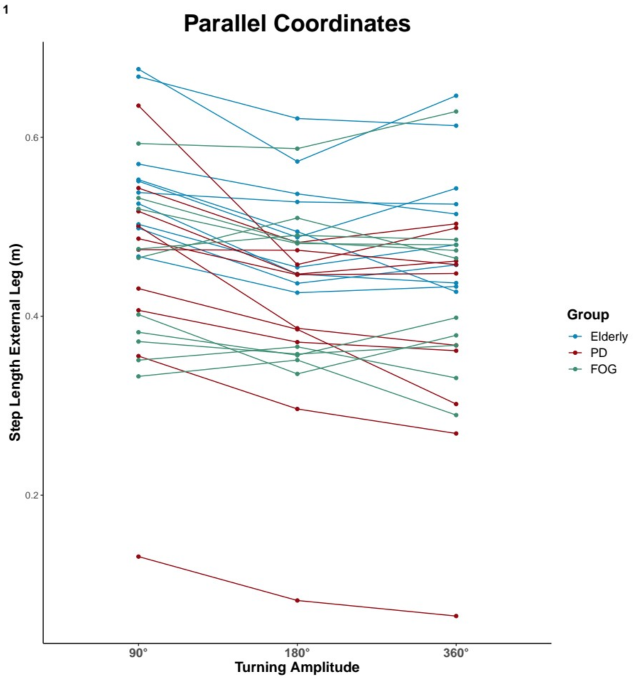


Figure 2

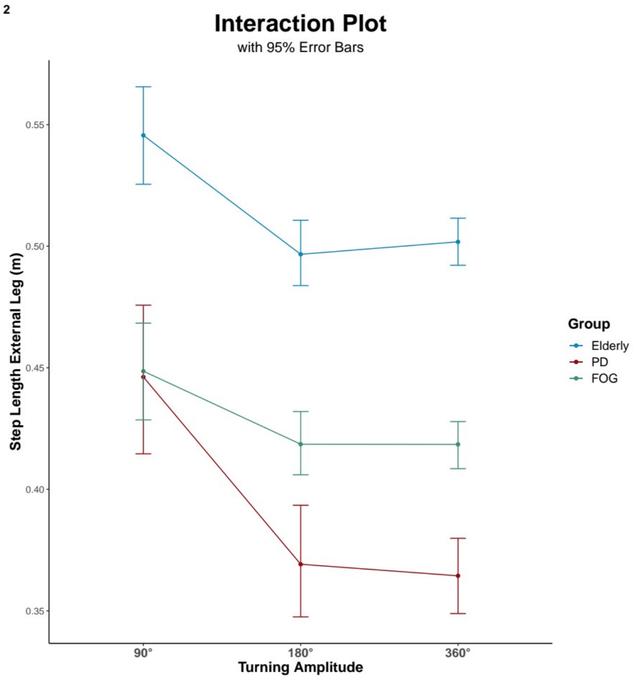


Figure 3

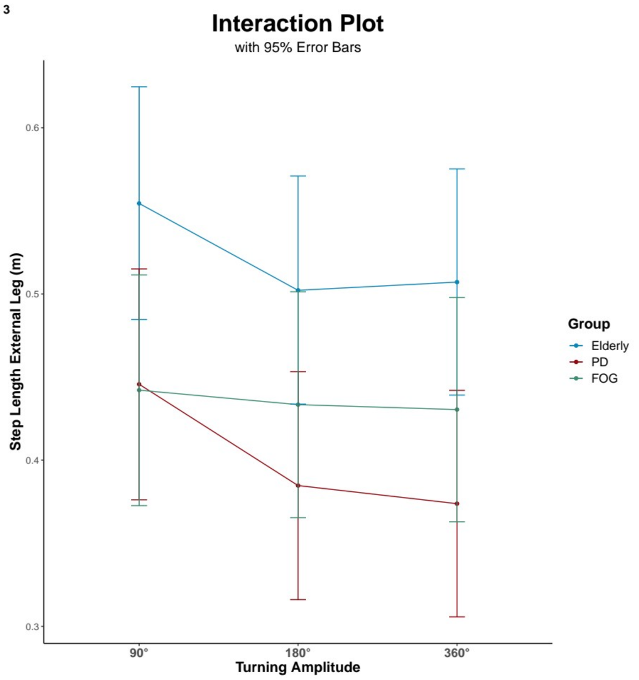

Figure 1

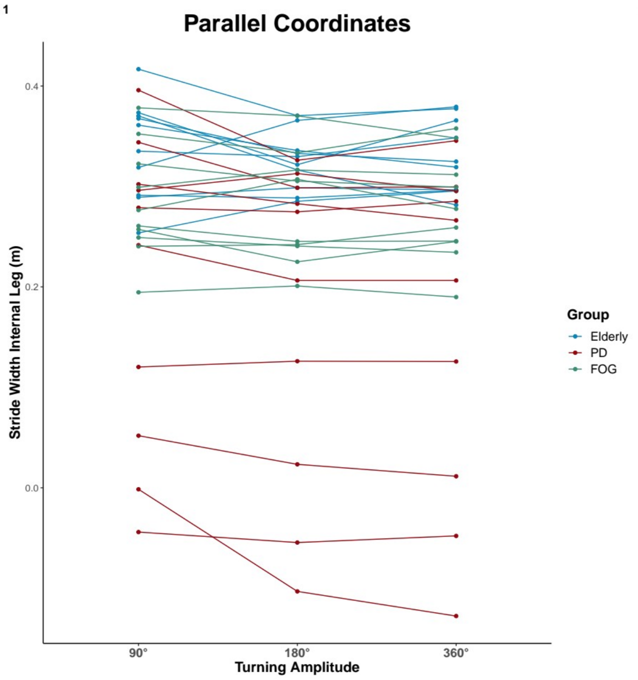


Figure 2

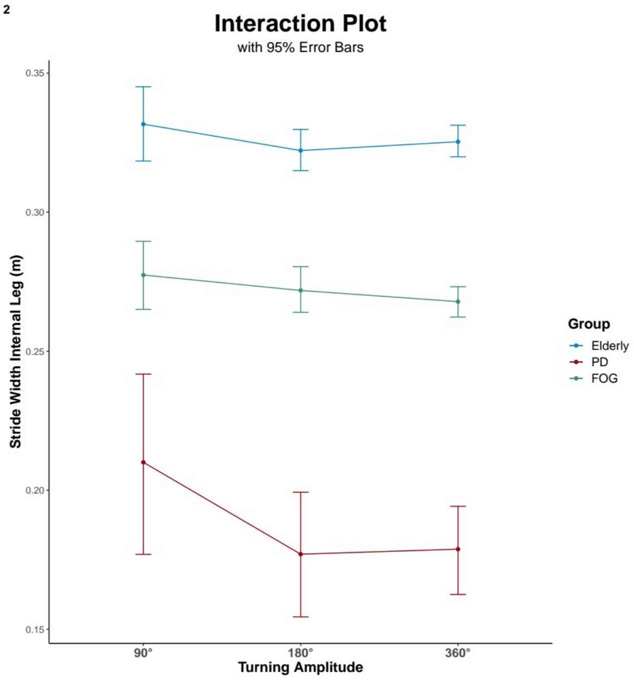


Figure 3

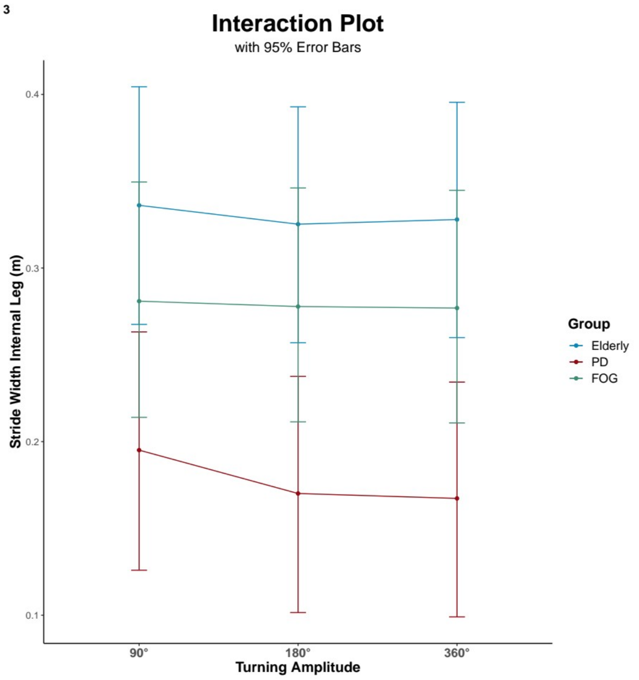

Figure 1

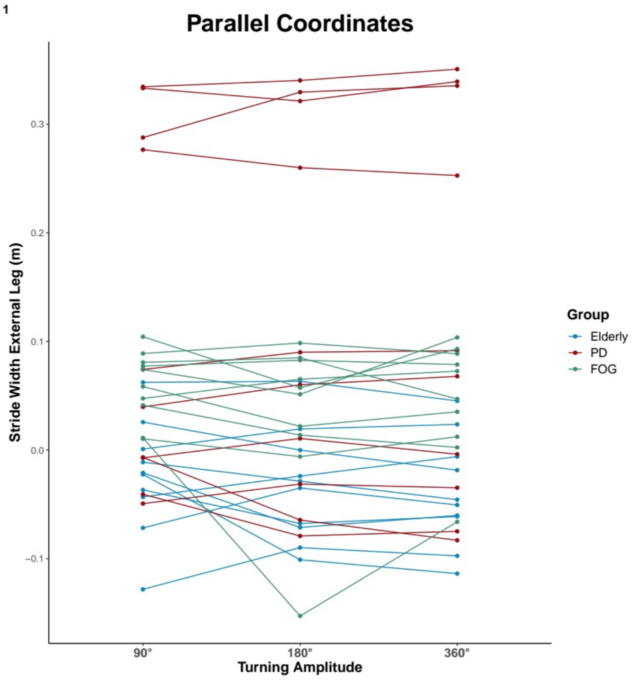


Figure 2

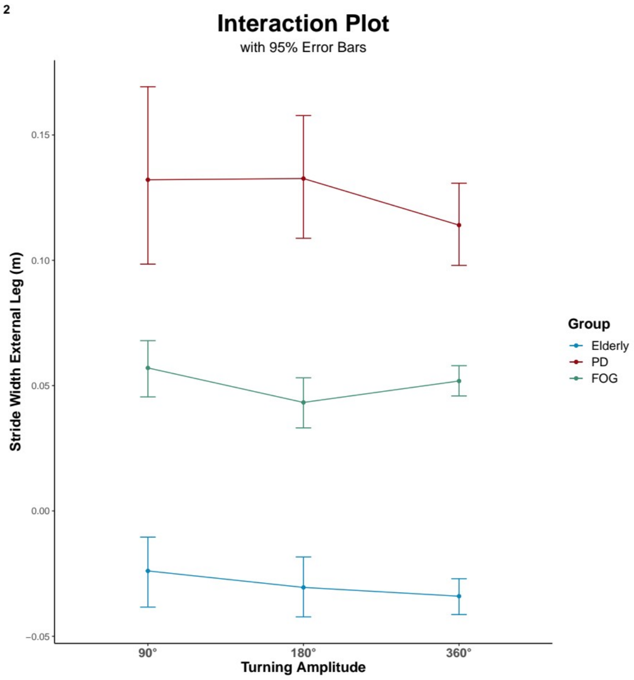


Figure 3

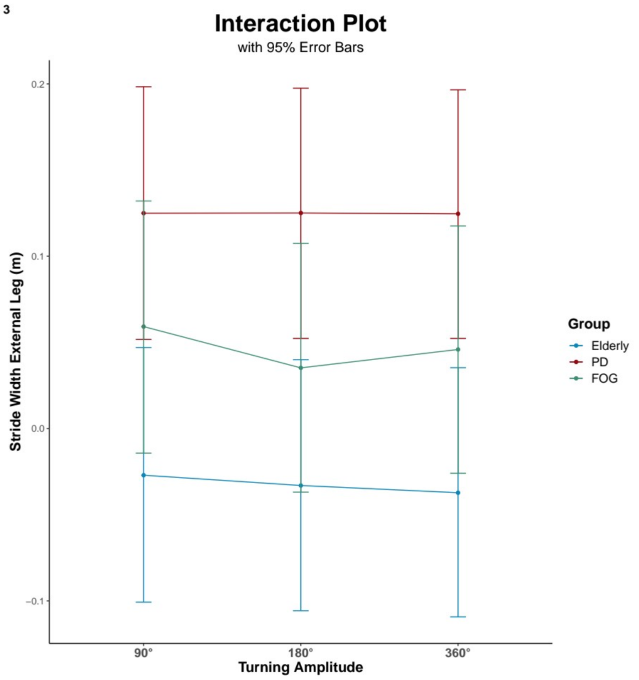

Figure 1

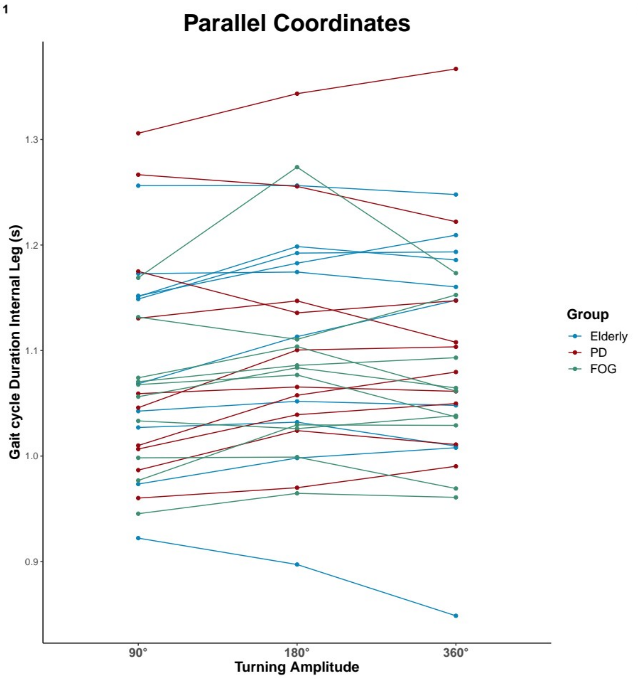


Figure 2

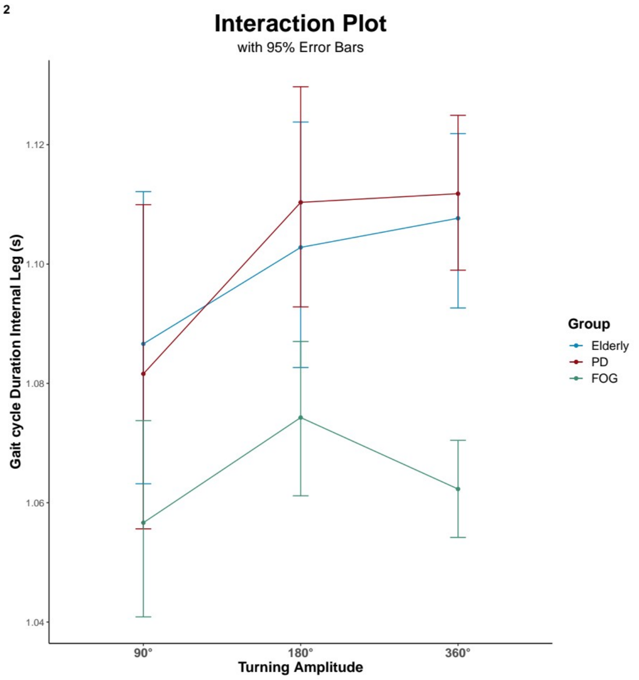


Figure 3

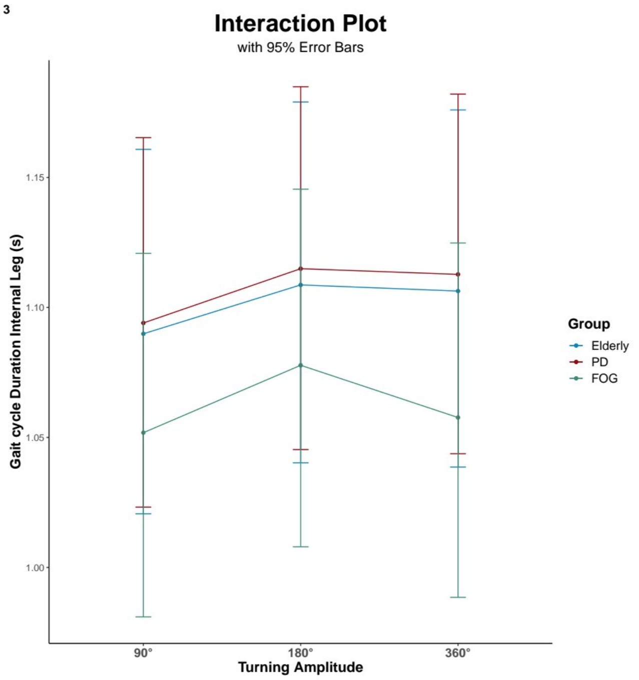

Figure 1

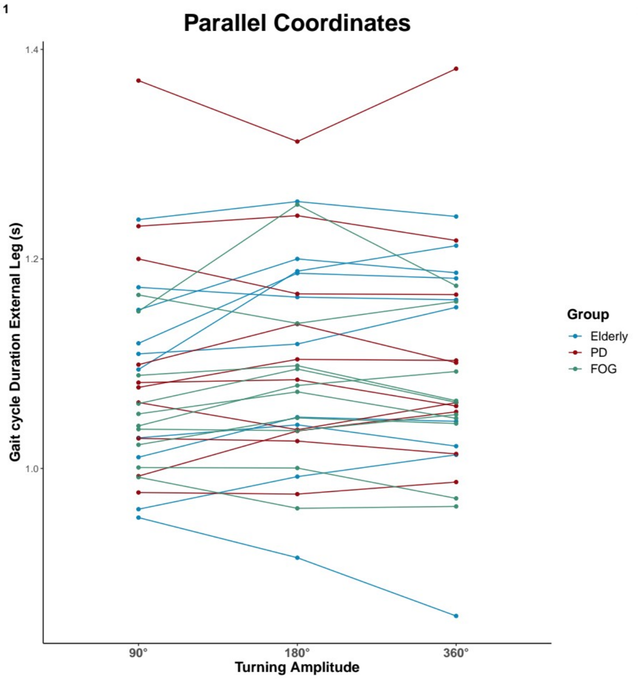


Figure 2

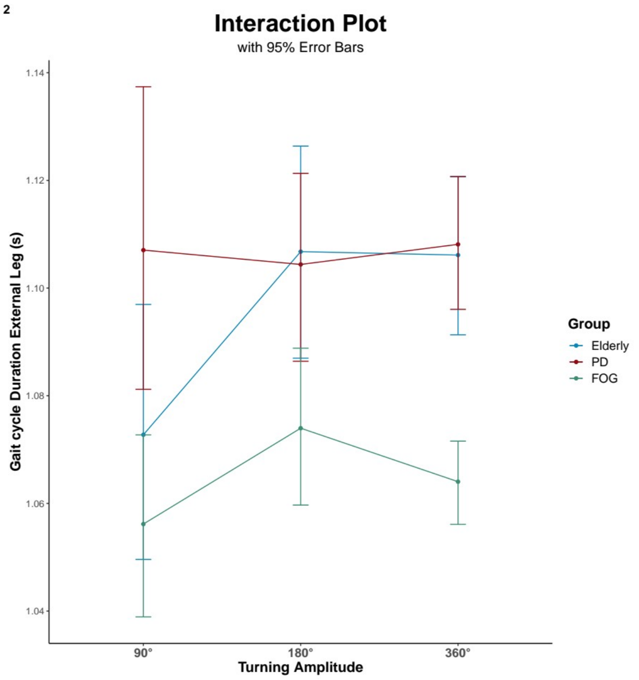


Figure 3

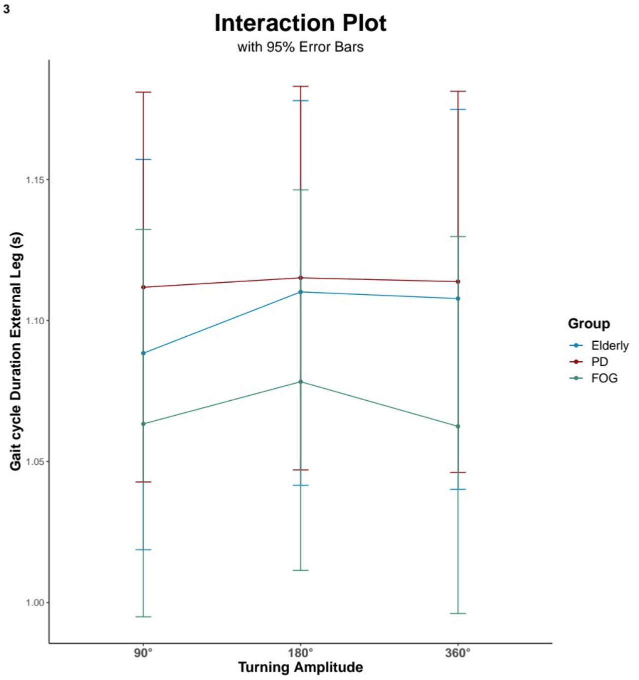

Figure 1

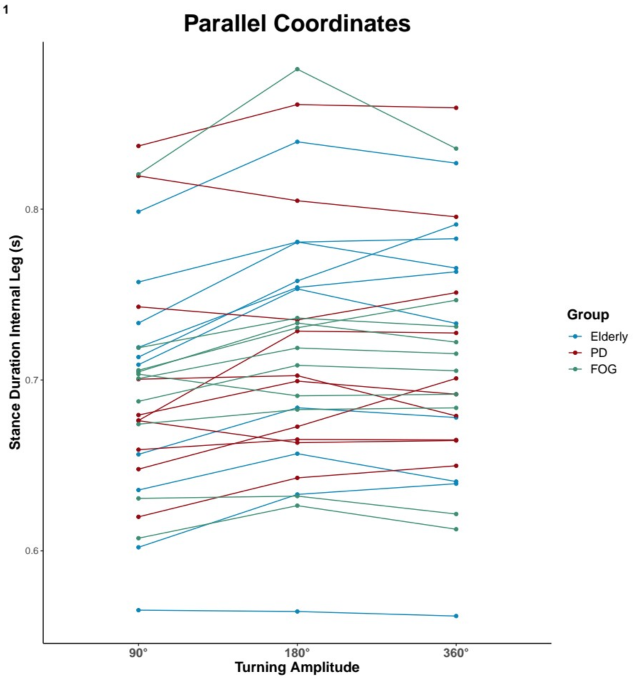


Figure 2

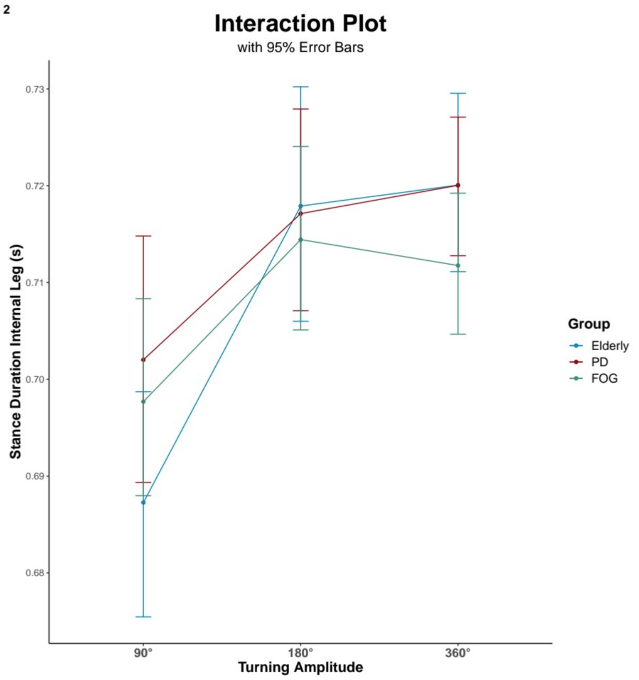


Figure 3

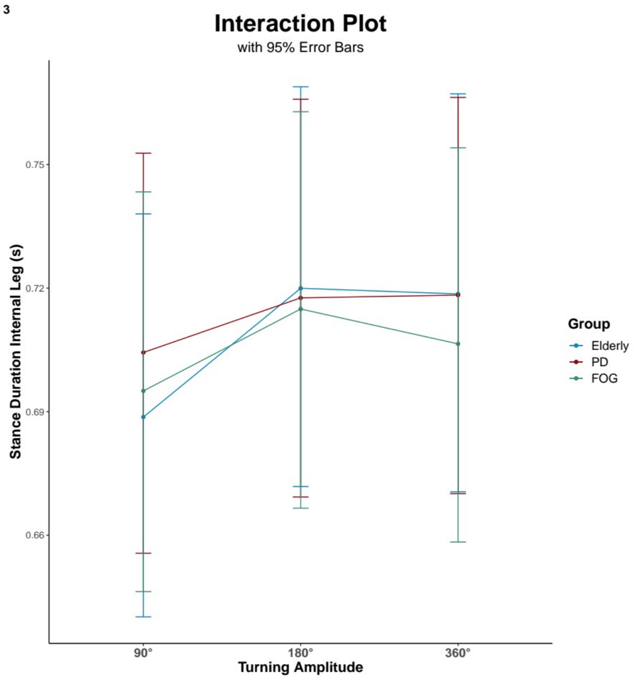

Figure 1

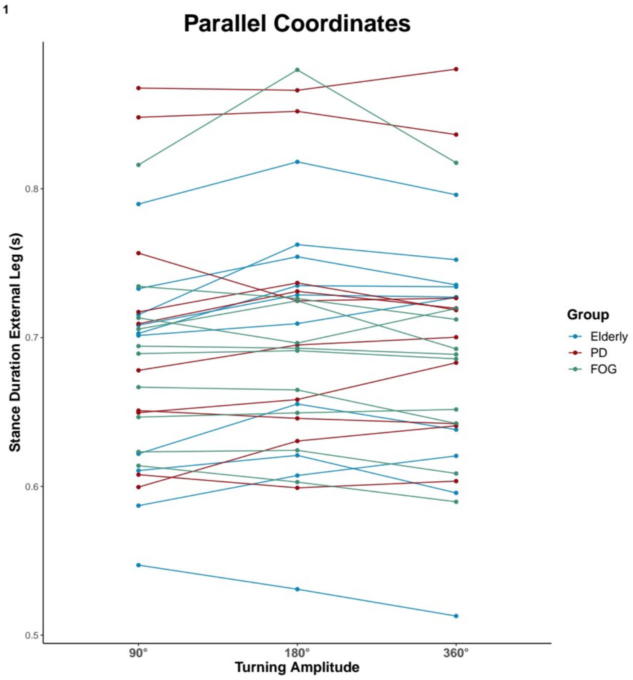


Figure 2

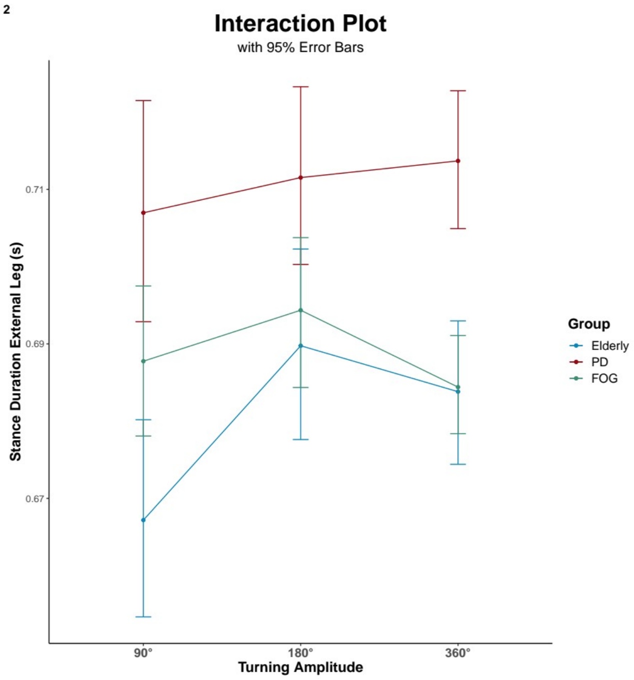


Figure 3

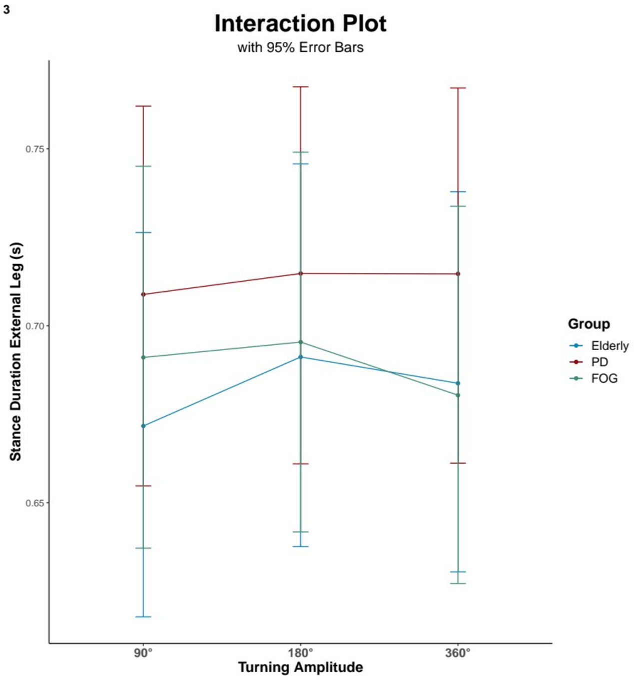

Figure 1

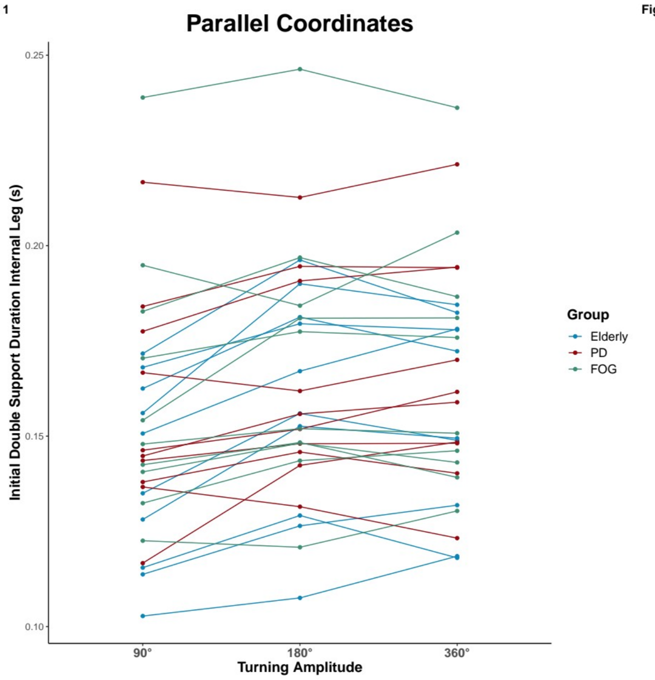


Figure 2

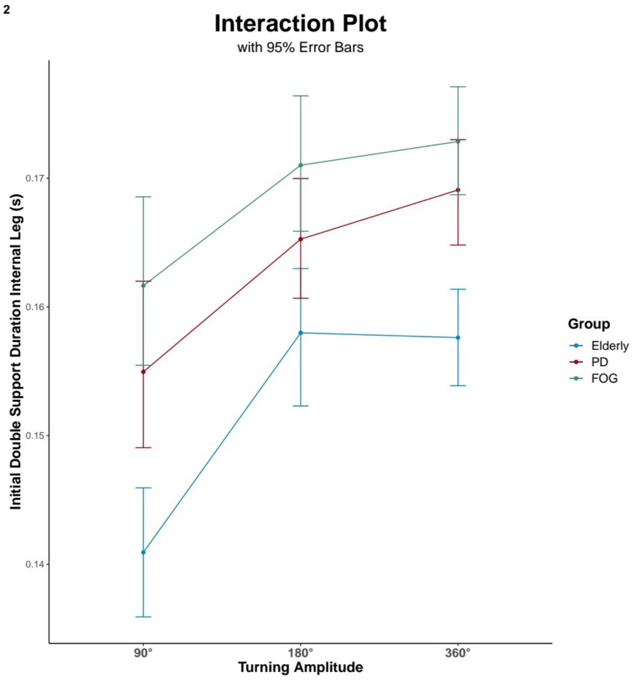


Figure 3

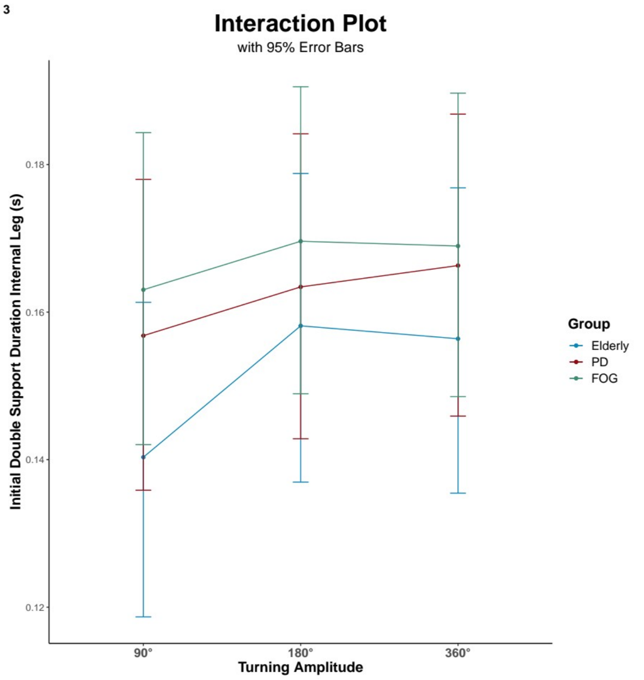

Figure 1

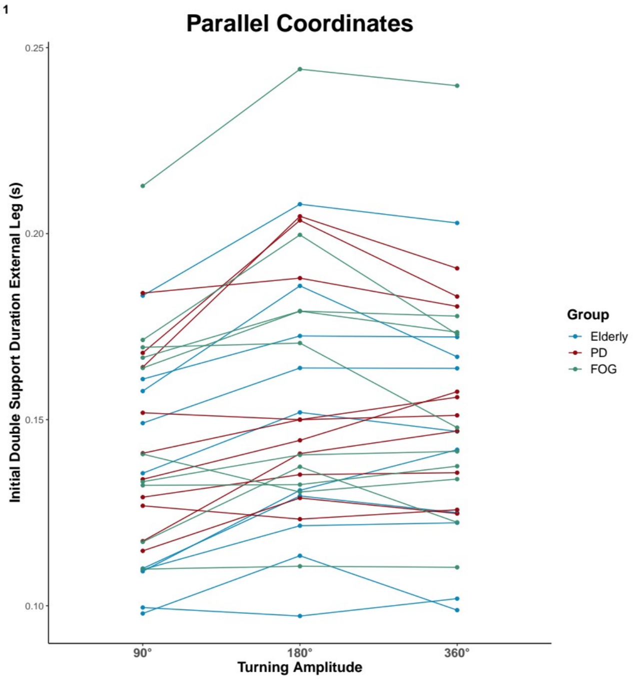


Figure 2

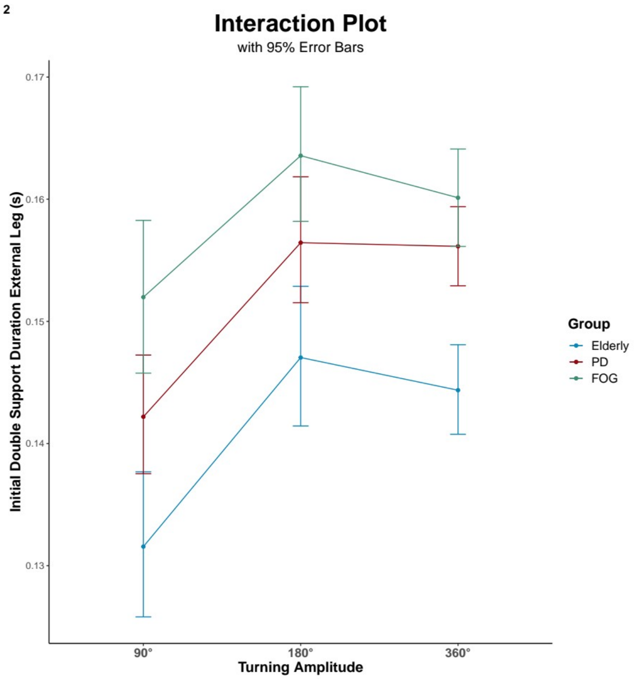


Figure 3

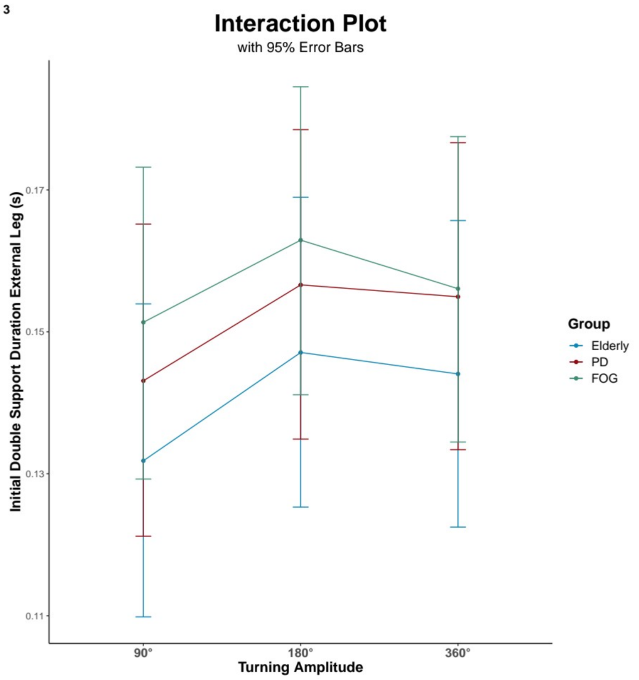

Figure 1

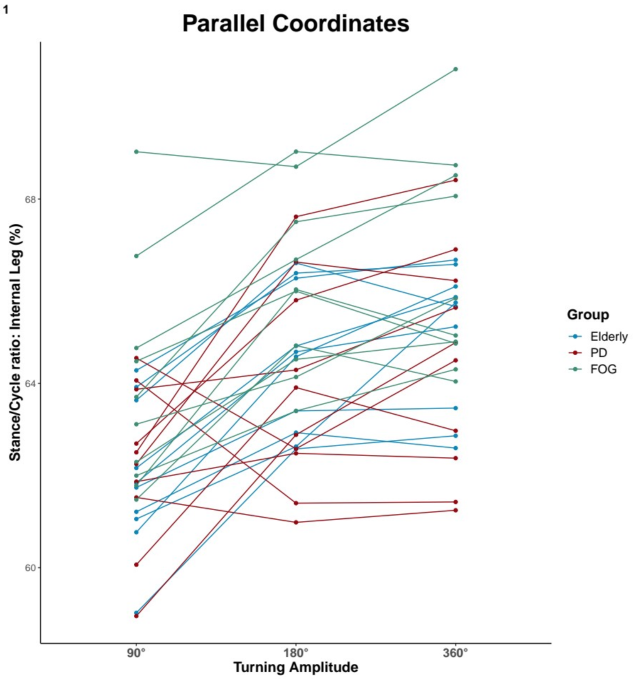


Figure 2

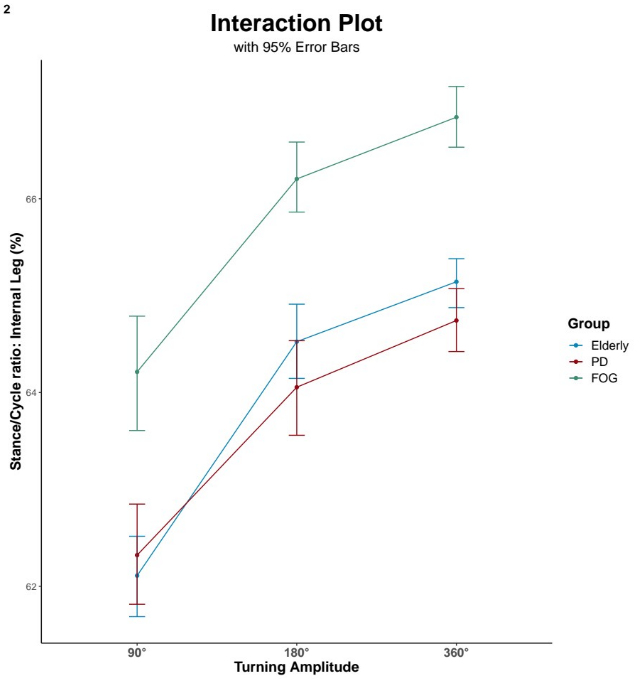


Figure 3

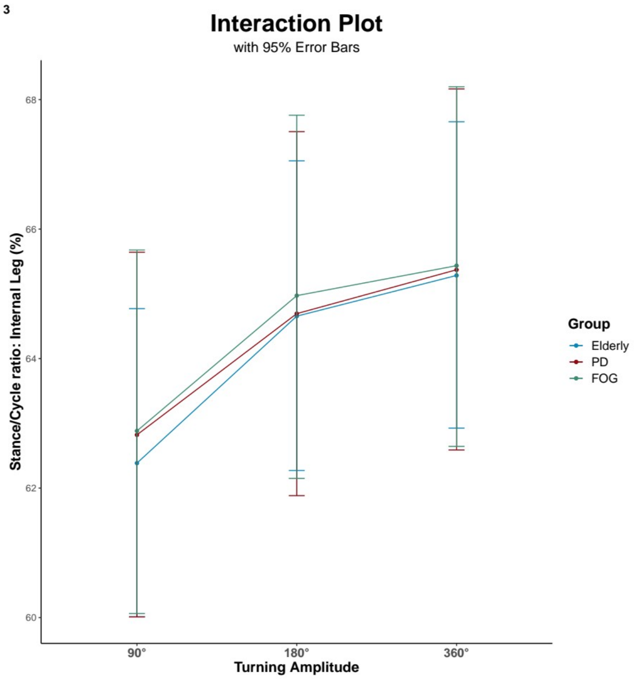

Figure 1

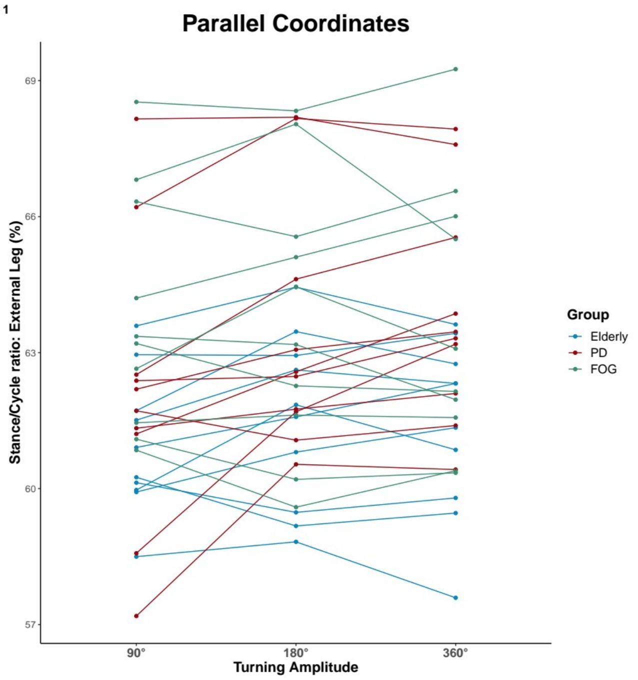


Figure 2

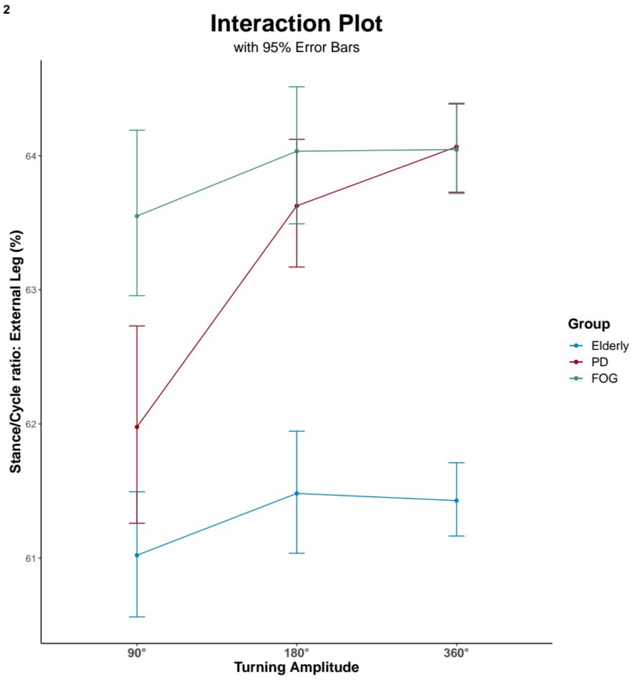


Figure 3

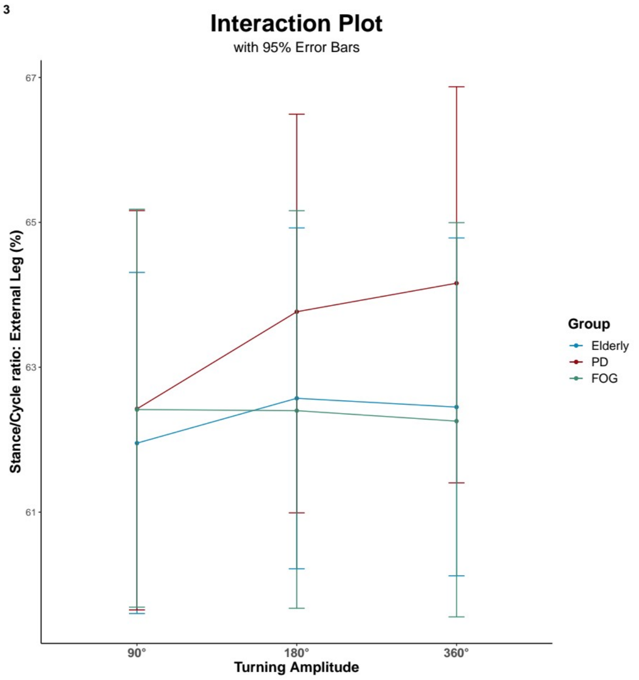

Supplement: Supplementary file 3 [file Table_2.docx]
